# The Society for Immunotherapy of Cancer consensus statement on immunotherapy for the treatment of non-small cell lung cancer (NSCLC)

**DOI:** 10.1186/s40425-018-0382-2

**Published:** 2018-07-17

**Authors:** Julie R. Brahmer, Ramaswamy Govindan, Robert A. Anders, Scott J. Antonia, Sarah Sagorsky, Marianne J. Davies, Steven M. Dubinett, Andrea Ferris, Leena Gandhi, Edward B. Garon, Matthew D. Hellmann, Fred R. Hirsch, Shakuntala Malik, Joel W. Neal, Vassiliki A. Papadimitrakopoulou, David L. Rimm, Lawrence H. Schwartz, Boris Sepesi, Beow Yong Yeap, Naiyer A. Rizvi, Roy S. Herbst

**Affiliations:** 1 Bloomberg Kimmel Immunotherapy Institute, Johns Hopkins Kimmel Cancer Center, Baltimore, MD 21231 USA; 2 Division of Oncology, Washington University, St Louis, MO 63110 USA; 3 Johns Hopkins School of Medicine, Baltimore, MD 21231 USA; 4 H. Lee Moffitt Cancer Center and Research Institute, Tampa, FL 33612 USA; 5 Johns Hopkins Kimmel Cancer Center, Baltimore, MD 21231 USA; 6 Yale Comprehensive Cancer Center, Yale University School of Nursing, New Haven, CT 06520 USA; 7 University of California Los Angeles Lung Cancer Research Program, David Geffen School of Medicine, University of California, Los Angeles, CA 90095 USA; 8 LUNGevity, Chicago, IL 60604 USA; 9 Department of Medicine, New York University, Perlmutter Cancer Center, NYU School of Medicine, New York, NY 10016 USA; 10 Division of Hematology-Oncology, Department of Medicine, David Geffen School of Medicine, University of California Los Angeles, Los Angeles, CA 90404 USA; 11 Department of Medicine, Memorial Sloan Kettering Cancer Center, New York, NY 10065 USA; 12 University of Colorado Denver School of Medicine, Denver, CO 80011 USA; 13 National Cancer Institute, Division of Cancer Treatment and Diagnosis, Cancer Therapy Evaluation Program, Rockville, USA; 14 Division of Oncology, Department of Medicine, Stanford University School of Medicine, Stanford, CA USA; 15 University of Texas MD Anderson Cancer Center, Houston, TX 77030 USA; 16 Department of Pathology, Yale University School of Medicine, New Haven, CT 06520 USA; 17 Department of Radiology, Columbia University College of Physicians and Surgeons and New York Presbyterian Hospital, New York City, NY 10032 USA; 18 Thoracic Surgical Oncology, University of Texas MD Anderson Cancer Center, Houston, TX 77030 USA; 19 Department of Medicine, Massachusetts General Hospital, Harvard Medical School, Boston, MA 02114 USA; 20 Columbia University Medical Center, New York, NY 10028 USA; 21 Yale Comprehensive Cancer Center, Yale School of Medicine, 333 Cedar Street, WWW221, New Haven, CT 06520-8028 USA

**Keywords:** Cancer immunotherapy, Consensus statement, Lung cancer, Non-small cell lung cancer, Guideline

## Abstract

Lung cancer is the leading cause of cancer-related mortality worldwide, with non-small cell lung cancer (NSCLC) accounting for over 85% of all cases. Until recently, chemotherapy – characterized by some benefit but only rare durable responses – was the only treatment option for patients with NSCLC whose tumors lacked targetable mutations. By contrast, immune checkpoint inhibitors have demonstrated distinctly durable responses and represent the advent of a new treatment approach for patients with NSCLC. Three immune checkpoint inhibitors, pembrolizumab, nivolumab and atezolizumab, are now approved for use in first- and/or second-line settings for selected patients with advanced NSCLC, with promising benefit also seen in patients with stage III NSCLC. Additionally, durvalumab following chemoradiation has been approved for use in patients with locally advanced disease. Due to the distinct features of cancer immunotherapy, and rapid progress in the field, clinical guidance is needed on the use of these agents, including appropriate patient selection, sequencing of therapies, response monitoring, adverse event management, and biomarker testing. The Society for Immunotherapy of Cancer (SITC) convened an expert Task Force charged with developing consensus recommendations on these key issues. Following a systematic process as outlined by the National Academy of Medicine, a literature search and panel voting were used to rate the strength of evidence for each recommendation. This consensus statement provides evidence-based recommendations to help clinicians integrate immune checkpoint inhibitors into the treatment plan for patients with NSCLC. This guidance will be updated following relevant advances in the field.

**Electronic supplementary material:**

The online version of this article (10.1186/s40425-018-0382-2) contains supplementary material, which is available to authorized users.

## Background

Lung cancer is associated with profound medical, psychosocial, economic, and societal burden. In the U.S. alone, an estimated 222,500 people will be diagnosed with lung cancer and approximately 155,870 people are expected to die of the disease in 2017 [1, 2]. Worldwide, lung cancer is the leading cause of cancer-related mortality, accounting for nearly 20% of all cancer-related deaths [3]. Non-small cell lung cancer (NSCLC) accounts for about 85% of all primary lung cancers, and most patients present with advanced, unresectable disease at the time of diagnosis [3, 4]. For several decades, cytotoxic chemotherapy was the only treatment that could prolong survival in patients with advanced NSCLC [4, 5]. However, advances in sequencing technology and increased understanding of tumor cell biology have led to the development of targeted therapies for NSCLC [5–7], including small molecule inhibitors for specific oncogenic driver alterations [8, 9]. Although these therapies have demonstrated efficacy in advanced NSCLC, resistance to targeted therapies remains inevitable [7, 10].

Cancer immunotherapy is a treatment modality used to mobilize the immune system to recognize and destroy cancer cells [11–13]. Immune checkpoint inhibitors have been developed to target self-tolerance pathways that are exploited by tumors to escape immune recognition and destruction [14, 15]. These agents act by modulating T cell function and have the potential to augment the host immune response against malignant cells [4, 6, 13, 16]. To date, four immune checkpoint pathway inhibitors have been approved by the United Stated Food and Drug Administration (FDA) for use in patients with NSCLC: nivolumab and pembrolizumab, both targeting the programmed cell death-1 (PD-1) receptor, as well as atezolizumab and durvalumab, targeting the anti-programmed death- ligand 1 (PD-L1) [9, 17]. Alongside these approvals, companion and complementary diagnostic assays measuring PD-L1 as a predictive biomarker in the tumor microenvironment have been approved to aid in patient selection [18]. However, variability in assay systems, tissue preparation and processing, and cutoff values have complicated the interpretation and consensus use of these assays [18–20].

The adoption of immunotherapy in routine clinical practice for NSCLC has come exceptionally quickly, starting from the first report of objective response to PD-1 blockade in 2012, to the first FDA approval in 2015 [21]. In this context, medical professionals who care for patients with NSCLC must keep pace with emerging evidence-based data, current practice guidelines, and new drug developments, to facilitate patient counseling and maximize clinical outcomes. In order to facilitate provider education, the Society for Immunotherapy of Cancer (SITC) established a Cancer Immunotherapy Guidelines Task Force for Lung Cancer (Additional file 1) charged with developing guidelines on the appropriate use of immunotherapy for the treatment of patients with lung cancer. The Task Force consisted of physician, physician assistant, and nurse practitioner experts in the management of patients with NSCLC, as well as a statistician and patient advocate. Five main topics were considered: (1) appropriate use of immune checkpoint blockade; (2) the role of PD-L1 biomarker testing in determining patient eligibility for treatment; (3) measuring and monitoring response to immune checkpoint inhibitors; (4) contraindications to treatment with immune checkpoint inhibitors; and (5) recognizing, monitoring, and managing immune-related adverse events (irAEs).

## Methods

### Consensus statement policy

The National Academy of Medicine’s (NAM, formerly the Institute of Medicine) March 2011 Standards for Developing Trustworthy Clinical Practice Guidelines [22] served as a model for organizing and preparing this consensus statement on the use of immunotherapy for the treatment of NSCLC. Previous SITC consensus guidelines on immunotherapy for the treatment of prostate carcinoma [23], renal cell carcinoma [24], hematologic malignancies [25], and cutaneous melanoma [26] served as models in the development of this consensus statement.

### Consensus panel

In April 2016, SITC convened a one-day meeting of multidisciplinary experts to develop consensus guidelines on the use of immunotherapy in patients with NSCLC. The consensus panel, which included SITC members and non-members, comprised 10 medical oncologists, 1 pulmonologist, 1 oncologist/pathologist, 1 lung cancer physician scientist, 1 thoracic surgeon, 2 pathologists, 1 radiologist, 1 statistician, 1 physician assistant, 1 nurse practitioner, and 1 patient advocate (Additional file 1). All panel members were based in the U.S. Four members – all medical oncologists – served on a Steering Committee tasked with leading the in-person meeting, guiding development of the manuscript and supplementary bibliography of NSCLC literature, and convening periodic conference calls with the wider panel to ensure that content kept pace with emerging data.

At the meeting, the consensus panel reviewed results from a previously distributed questionnaire that solicited information on their practice using FDA-approved agents to treat patients with NSCLC. A post-meeting questionnaire (May 2017) and follow-up survey questions (February 2018) were circulated to the consensus panel to ensure that the final guideline recommendations reflected the most recent clinical trial data, drug approvals, and clinical experience. Due to differences in drug approval, availability and regulations between countries, discussions focused solely on agents approved by the FDA for the treatment of patients in the U.S., and on issues pertaining to U.S-based clinical practice.

This statement represents expert consensus on the management of patients with NSCLC. The recommendations of the consensus panel, as set forth in this manuscript, are intended to provide guidance and should not be used as a substitute for the individual professional judgment of the treating physician. The full version of this and other consensus statements can be found on the SITC website [27].

### Disclosures and conflicts of interest

All members of the consensus panel disclosed potential conflicts of interest using the SITC disclosure form, which mandates full financial or other disclosures including relationships with regulatory or commercial entities that might reasonably be expected to have a direct impact on, or benefit from, the document. No commercial funding was used to support the consensus panel, literature review, or preparation of the manuscript. The final version of this consensus statement was made available to the entire SITC membership during an open comment period (Additional file 2).

### Literature search

A search of the medical literature was executed using MEDLINE and PubMed databases, to develop a comprehensive bibliography of literature pertaining to immunotherapy in NSCLC. The main MeSH search phrase – non-small cell lung cancer– was paired with other search terms including nivolumab, ipilimumab, pembrolizumab, durvalumab, atezolizumab, vaccines, PD-L1/PD-1, immunotherapy, combination immunotherapy, and immunotherapy adverse events. The search, which was limited to clinical trials, meta-analyses, practice guidelines in humans, randomized controlled trials, controlled clinical trials, and clinical studies, includes articles published between January 1, 2008 and February 12, 2018. After removing duplicates, reviewing articles for accuracy, and supplementing the literature search with additional articles identified as relevant by the Task Force, a 151-item bibliography was finalized (Additional file 3).

The literature was graded according to a previously established rating system in which Level A represents strong evidence-based data derived from prospective, randomized clinical trials and meta-analyses; Level B represents moderately supported data derived from uncontrolled, prospective clinical trials; and Level C represents weak supporting data derived from reviews and case reports [26].

### Consensus recommendations

#### Clinical question 1: What is the appropriate use of immune checkpoint blockade in patients with NSCLC?

Over half of patients newly diagnosed in the U.S. with NSCLC present with advanced disease that has already metastasized [2]. At this stage, there have historically been no curative treatment options and few patients (< 5%) survived five or more years [2]. However, there are several treatment options available that can prolong survival in patients with metastatic disease. The Task Force considered the following immunotherapy options for patients with advanced disease: pembrolizumab as a single agent in the first-line setting; nivolumab, pembrolizumab, or atezolizumab in the second-line setting; pembrolizumab in combination with carboplatin and pemetrexed in the first-line setting; and durvalumab in the maintenance/adjuvant setting. Additionally, the Task Force considered durvalumab following chemoradiation in patients with locally, advanced disease.

### Initial assessment

In order to determine eligibility for these agents, patients with advanced NSCLC should undergo a comprehensive diagnostic workup, including a complete review of clinical, radiological, and pathological information. This workup should include determination of tumor histological subtype, and molecular analysis to identify targetable driver mutations. The Task Force was in agreement that the analysis of PD-L1 expression by an immunohistochemistry (IHC)-based test to determine PD-L1 expression levels should be routine for all patients with newly diagnosed advanced NSCLC. Prior to initiation of immunotherapy, tests recommended by the majority of the Task Force included computerized tomography (CT) of the chest, abdomen, and pelvis (88% recommended) and thyroid function tests (81%).

The Task Force did not reach a majority in recommending tests including creatinine clearance (50%); magnetic resonance imaging (MRI) of the brain (50%); and pulmonary function tests (50%). Of note, National Comprehensive Cancer Network (NCCN) guidelines for the treatment of NSCLC agree with the Task Force recommendation for CT scans of all patients, but differ by recommending brain MRI across all disease stages. NCCN only recommends pulmonary function tests in specific cases and if surgery is an option [7].

### Literature review and analysis

#### Nivolumab

In two large, international phase III trials of patients with advanced squamous or non-squamous NSCLC whose disease had progressed on platinum-based chemotherapy, nivolumab, a fully humanized IgG4 monoclonal antibody against PD-1, dosed at 3 mg/kg every 2 weeks, demonstrated improved survival over docetaxel [28, 29]. In the trials of squamous cell NSCLC, nivolumab improved median overall survival (OS) in 272 patients with previously treated, advanced squamous cell NSCLC (OS: 9.2 vs. 6.0 months; hazard ratio [HR] 0.59; 95% confidence interval [CI]: 0.44–0.79; *p* < 0.001) [28]. In non-squamous NSCLC, nivolumab demonstrated superior median OS versus docetaxel in 582 patients (OS: 12.2 months versus 9.4 months; HR 0.73, 95% CI: 0.60–0.89; *p* = 0.002) [29]. In these two trials, treatment-related AEs grade ≥ 3 were reported in ≤10% of patients receiving nivolumab compared with ~ 55% of those in the docetaxel group [28, 29]. Based on these results, nivolumab was approved by the FDA, at a dose of 240 mg IV every 2 weeks, for patients with previously-treated, metastatic squamous (March 2015) and non-squamous (October 2015) cell lung carcinoma who have progressed on platinum-containing therapy [30]. Additionally, a fixed dose schedule of nivolumab at 480 mg IV every 4 weeks was recently approved by the FDA for use in all previously-approved indications to treat patients with NSCLC [30].

Nivolumab was also tested against standard platinum-doublet chemotherapy in the first-line setting, in a randomized phase III study in 541 treatment-naïve patients with advanced PD-L1 positive (≥ 5% per IHC 28–8 pharmDx assay) NSCLC [31]. However, this study did not reach its primary endpoints: neither progression-free survival (PFS) nor OS were improved with nivolumab compared to platinum-based chemotherapy, even in the PD-L1 ≥ 50% positive group.

Results from the CheckMate 227 phase III clinical trial indicate that patients with advanced NSCLC – squamous and non-squamous – and high tumor mutational burden (TMB, measured with the FoundationOne CDx™ assay) had increased PFS when treated with first-line combination nivolumab + ipilimumab compared to chemotherapy, regardless of tumor PD-L1 expression (HR 0.58; 97.5%CI: 0.41–0.81; *p* < 0.001). Recently presented data from this study also indicate that patients with advanced NSCLC treated with nivolumab + chemotherapy also had increased median PFS compared to patients treated with chemotherapy alone (5.6 mos vs 4.7 mos, respectively; HR = 0.74 [95% CI: 0.58–0.94]) [32]. CheckMate 227 also includes cohorts to compare combination nivolumab + ipilimumab efficacy against nivolumab monotherapy, but these data are not mature at the time of writing [33]. The CheckMate 227 trial is ongoing and FDA approvals for combination nivolumab + ipilimumab and nivolumab + chemotherapy have not yet been granted for the treatment of patients with advanced NSCLC.

#### Pembrolizumab

Pembrolizumab, a fully humanized IgG4 monoclonal antibody against PD-1, was first tested at a dose of 2 mg/kg or 10 mg/kg every 3 weeks, or 10 mg/kg every 2 weeks, in a large phase I dose expansion trial in 495 patients with advanced NSCLC [34]. The reported overall response rate (ORR) in this study was 19.4% (95% CI: 16–23.2), with response rates of 18% (95% CI: 14.4–22.2) in 394 patients with previously treated disease, and 24.8% (95% CI: 16.7–34.3) in 101 patients with untreated disease [34]. In this study, patients with intermediate or high PD-L1 expression (defined as tumor proportion score [TPS] ≥ 1% or ≥ 50%, respectively, using the anti-PD-L1 antibody clone 22C3 in an IHC assay) had improved outcomes compared to those with no PD-L1 expression. An additional 55 patients enrolled in the study were not part of the primary efficacy analysis, but results in this supplementary cohort confirmed efficacy at 2 mg/kg. A subsequent phase II/III study in 1034 previously treated patients with a PD-L1 TPS ≥ 1% compared pembrolizumab (2 mg/kg or 10 mg/kg) with docetaxel. Median OS was significantly longer for pembrolizumab 2 mg/kg (HR 0.71, 95% CI: 0.58–0.88; *p* = 0.001) and 10 mg/kg (HR·0.61, 95% CI: 0.49–0.75; *p* < 0.001) versus docetaxel. Moreover, grade ≥ 3 adverse events were less common with pembrolizumab [35]. Based on these data, on October 2, 2015 the FDA approved the use of pembrolizumab, at a dose of 200 mg IV every 3 weeks, to treat patients with PD-L1-positive (TPS ≥ 1%, as determined by an FDA-approved test), metastatic NSCLC whose disease has progressed on or following platinum-containing chemotherapy and disease progression on FDA-approved therapy for patients with EGFR or ALK genomic tumor aberrations [36].

These results paved the way for a phase III trial comparing pembrolizumab monotherapy with platinum-doublet chemotherapy in the first-line setting [37]. In this open-label study, 305 patients with untreated advanced NSCLC, no actionable EGFR or ALK mutations, and PD-L1 TPS score ≥ 50% were randomized to receive pembrolizumab (200 mg fixed dose every 3 weeks) or investigator’s choice of platinum-based chemotherapy. Pembrolizumab demonstrated significantly longer PFS (median 10.3 vs. 6.0 months; HR 0.5, 95% CI: 0.37–0.68, *p* < 0.001) and a higher rate of OS at 6 months compared with chemotherapy [80.2% vs. 72.4% (HR 0.6, 95% CI: 0.41–0.89, *p* = 0.005)] [37]. Based on these results, on October 24, 2017 the FDA expanded the approval of pembrolizumab to include first-line treatment of patients with high PD-L1-expression (TPS ≥ 50%, as determined by an FDA-approved test) metastatic NSCLC with no EGFR or ALK genomic aberrations.

In a randomized, phase II study pembrolizumab was also tested as first-line therapy in combination with chemotherapy (in this instance, carboplatin and pemetrexed), versus chemotherapy alone, in 123 treatment-naive patients with metastatic NSCLC, independent of PD-L1 expression. In this trial, pembrolizumab plus chemotherapy demonstrated an ORR nearly double that of chemotherapy alone (55% [95% CI: 42–68] vs. 29% [95% CI: 18–41]; *p* = 0.0032). The addition of pembrolizumab led to improvement in PFS (HR 0.53, 95% CI: 0.31–0.91; *p* = 0.02), with a median PFS of 13.0 months (95% CI: 8.3-not estimable) for patients treated with pembrolizumab plus chemotherapy compared to 8.9 months (95% CI: 4.4–10.3) with chemotherapy alone [38]. There was an increase in grade 3/4 treatment-related adverse events with the addition of pembrolizumab (39% vs. 26%), the most common being anemia, neutropenia, and sepsis. No statistically significant OS benefit was reported. These results nevertheless led to the accelerated approval of pembrolizumab in combination with carboplatin and pemetrexed for first-line treatment of patients with non-squamous NSCLC, independent of PD-L1 status, on May 10, 2017. Subsequent data from this trial at a median follow-up of 18.7 months demonstrated ongoing, statistically significant benefits to PFS (19.0 vs 8.9 months; HR 0.54 [95% CI: 0.33–0.88; *p* = 0.007] and ORR (57 vs. 32% [95% CI: 7–41%]; p = 0.003) with the pembrolizumab/chemotherapy combination [39]. HR for OS using the combination showed ongoing improvement (HR 0.59 [95% CI: 0.34–1.05; *p* = 0.03]) and median OS had not been reached at the time of data cutoff (vs. 20.9 months with chemotherapy alone). Grade ≥ 3 treatment-related AEs occurred in 41% vs. 29% of patients in the pembrolizumab/chemotherapy vs. chemotherapy only arms, respectively. These data were verified in a recently reported phase III trial, indicating that first-line pembrolizumab plus chemotherapy (pemetrexed plus cisplatin or carboplatin) in patients with advanced or metastatic NSCLC, irrespective of PD-L1 expression, reduced risk of death by 51% at median follow up (10.5 months) compared to patients receiving doublet chemotherapy (HR 0.49; 95% CI: 0.38–0.64; *p* < 0.001) [40].

Pembrolizumab has also been tested in combination with carboplatin + nab-paclitaxel/paclitaxel as first-line treatment in patients with advanced, squamous cell NSCLC. Recent data from the phase III KEYNOTE-407 clinical trial demonstrated increased OS at median follow-up (7.8 months) in patients who were treated with combination pembrolizumab + chemotherapy compared to patients treated with chemotherapy alone (HR = 0.64, 95% CI: 0.49–0.85, *p* = 0.0008). Additionally, OS benefit provided by combination pembrolizumab + chemotherapy was observed in patients regardless of tumor PD-L1 status (TPS < 1%, HR = 0.61 [95% CI: 0.35–0.98]; TPS 1–49%, HR = 0.57 [95% CI: 0.36–0.90]; TPS > 50%, HR = 0.64 [95% CI: 0.37–1.10]). Grade 3–5 AEs were comparable across the pembrolizumab/chemotherapy and placebo cohorts (69.8% vs. 68.2%, respectively) [41]. Based on these data, a supplemental Biologics License Application concerning combination pembrolizumab + chemotherapy for the treatment of patients with advanced, squamous cell NSCLC was submitted to the FDA in May 2018, and is under review as of this writing.

#### Atezolizumab

Atezolizumab, a humanized monoclonal IgG1 antibody against PD-L1, was shown in two open-label, phase II studies to have clinical activity in patients with NSCLC. In the first study, 659 chemotherapy-naïve and previously treated patients with PD-L1-positive (TPS ≥ 5% on tumor or infiltrating immune cells) NSCLC were treated with atezolizumab in three different cohorts [42]. Cohort 1 comprised chemotherapy-naïve patients, while cohorts 2 and 3 had received prior treatment (second-line or third- and beyond, respectively). Atezolizumab met its primary objective of ORR vs. historical controls: at the 12 month follow-up, ORR was 18–22% for the three cohorts, with median OS of 23.5 months, 15.5 months, and 13.2 months in cohorts 1–3, respectively. PD-L1 expression levels were modestly associated with clinical benefit [42]. In the second study, atezolizumab was found to be superior to docetaxel in 284 patients with previously treated advanced or metastatic NSCLC: OS in the intention-to-treat population was 12.6 months for atezolizumab versus 9.7 months for docetaxel (HR 0.73, 95% CI 0.53–0.99; *p* = 0.04) [43]. These studies led to the approval of atezolizumab at a dose of 1200 mg IV every 3 weeks for patients with previously treated metastatic NSCLC regardless of PD-L1 expression, on October 18, 2016 [44]. The results were confirmed in a randomized, phase III, multicenter trial that compared atezolizumab with docetaxel in 850 patients who had progressed following one or more platinum-containing combination regimens [45]. OS (primary endpoint) was longer with atezolizumab in the intent-to-treat population, with a median OS of 13.8 months vs. 9.6 months (HR 0.74, 95% CI: 0.63–0.87; *p* = 0.0004) [45].

In April 2018, data from the IMpower 150 phase III clinical trial indicate that patients with advanced, non-squamous NSCLC – including patients with a sensitizing EGFR mutation or ALK translocation with disease progression, or intolerance of treatment with one or more approved targeted therapies – treated with quadruplet therapy including atezolizumab, doublet chemotherapy, and anti-vascular endothelial growth factor A (VEGF-A) bevacizumab. Patients receiving quadruplet therapy had improved median PFS compared to patients receiving combination bevacizumab + chemotherapy (8.3 mos vs. 6.8 mos, respectively. HR 0.62; 95% CI: 0.52–0.74, *p* < 0.0001). PFS benefits were irrespective of patient tumor PD-L1 status [46]. This trial is ongoing and this regimen is not presently FDA approved for the treatment of patients with advanced NSCLC.

Recent data from the phase III IMpower 131 trial indicate that first-line atezolizumab + carboplatin & nab-paclitaxel increased PFS in patients with advanced squamous NSCLC compared to the chemotherapy doublet (6.3 mos vs 5.6 mos, respectively; HR = 0.71 [95% CI: 0.60–0.85], *p* < 0.0001). In patients with high-PD-L1, OS benefit from atezolizumab + chemotherapy was observed compared to patients treated with chemotherapy (23.6 mos vs 14.1 mos, HR = 0.56 [95% CI: 0.32–0.99]. In PD-L1 low or negative subgroups, however, no OS difference was observed between patients treated with atezolizumab + chemotherapy or chemotherapy alone (PD-L1 low: 12.4 mos vs 16.6 mos, HR = 1.34 [95% CI: 0.95–1.90]; PD-L1 negative: 13.8 mos vs 12.5 mos, HR = 0.86 [95% CI: 0.65–1.15]) [47]. This trial is ongoing.

#### Durvalumab

Late stage clinical trial results have recently been reported for durvalumab, a human IgG1 monoclonal antibody against PD-L1, in patients with locally advanced, unresectable stage III NSCLC [48]. In this randomized, phase III study, 713 patients received durvalumab (*n* = 476) or placebo (*n* = 237) as consolidation therapy following chemoradiation. Median PFS was significantly longer in patients who received durvalumab compared with placebo (16.8 vs. 5.6 months; HR 0.52; 95% CI: 0.42–0.65; *p* < 0.001), with an ongoing PFS rate advantage at 18-months (44.2% vs. 27.0%; *p* < 0.0001). Results were consistent across pre-specified demographic and clinical subgroups, including never-smokers, irrespective of baseline PD-L1 tumor expression. In addition, durvalumab illustrated superior outcomes for secondary endpoints including overall response rate (26% vs. 14%; *p* < 0.001) and median duration of response (72.8% vs. 46.8% at 18 months). The incidence of grade 3/4 adverse events was similar with durvalumab (29.9%) and placebo (26.1%), although a higher proportion of patients taking durvalumab discontinued treatment as a result (15.4% vs. 9.8%). OS results from this study remain immature. On February 16th, 2018, durvalumab gained approval at a dose of 10 mg/kg IV every 2 weeks, for a maximum of 1 year, for patients with locally advanced, unresectable NSCLC whose disease has not progressed following chemoradiotherapy.

### Consensus management of stage III NSCLC

#### Maintenance/adjuvant therapy

A majority of the task force agreed that durvalumab should be used in stage III patients who have not progressed post-chemoradiation, based on Level A evidence. Limited data is available concerning durvalumab duration, and this recommendation will be reassessed as more results become available.

### Consensus management of metastatic NSCLC

#### First-line therapy

**Fig. 1:**
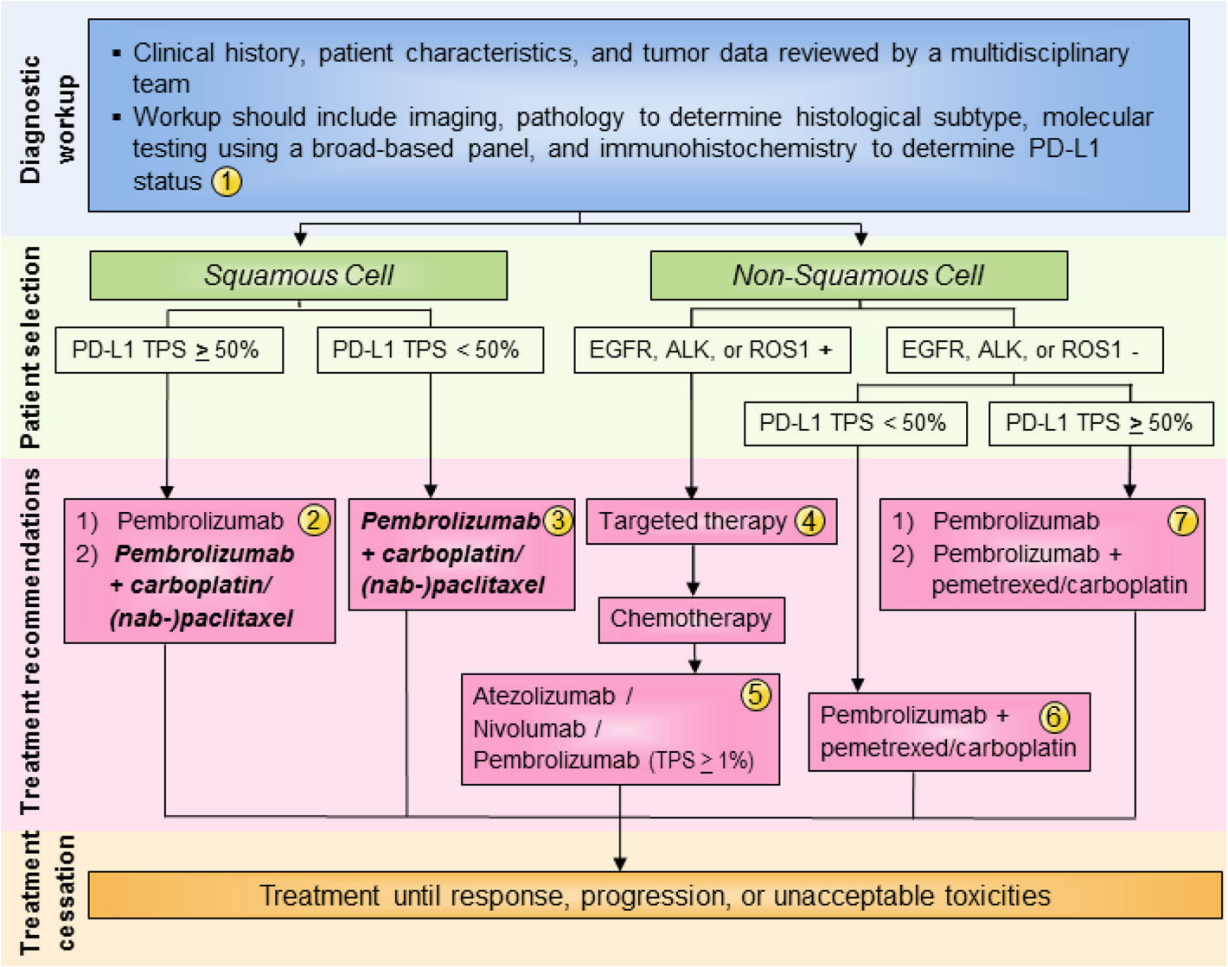
Advanced/metastatic NSCLC treatment algorithm. All treatment options shown may be appropriate and final selection of therapy should be individualized based on patient eligibility, prior treatment, and treatment availability at the treating physician’s discretion. These algorithms represent consensus sequencing suggestions by the panel. (1) All patients should be evaluated by a multidisciplinary team to determine histological subtype, identify targetable driver mutations, and perform PD-L1 testing. The Task Force unanimously agreed that all newly diagnosed patients should receive testing for PD-L1. (2) For patients with squamous NSCLC with TPS ≥ 50%, the Task Force supports pembrolizumab monotherapy first-line. When FDA approval is granted, the Task Force also supports pembrolizumab in combination with carboplatin & (nab-) paclitaxel in specific cases. (3) When approved by the FDA, the Task Force recommends combination pembrolizumab + pemetrexed & (nab-) paclitaxel first-line in patients with squamous histology and PD-L1 TPS < 50%. (4) In patients with non-squamous cell NSCLC tumors positive for EGFR, ALK, or ROS1 aberrations, appropriate targeted therapy should be administered. (5) Patients with squamous or non-squamous cell NSCLC who have progressed on platinum-containing chemotherapy and who have not previously received a checkpoint inhibitor should be considered for atezolizumab, nivolumab, or pembrolizumab. (6) The Task Force unanimously agreed that patients with non-squamous cell NSCLC without EGFR, ALK, or ROS1 aberrations and TPS < 50% should receive combination pembrolizumab + pemetrexed & carboplatin. (7) In patients with non-squamous cell NSCLC without EGFR, ALK, or ROS1 aberrations and TPS ≥ 50%, the Task Force recommends pembrolizumab monotherapy, but recognizes that combination pembrolizumab + pemetrexed & carboplatin can be appropriate in specific cases

The Task Force unanimously agreed that pembrolizumab should be used first-line in patients with PD-L1-positive (TPS ≥ 50%) non-squamous metastatic NSCLC (Fig. 1), based on Level A evidence. The Task Force notes that first-line pembrolizumab plus pemetrexed and carboplatin may also be appropriate for patients with non-squamous histology and PD-L1 TPS ≥ 50% in a case-by-case basis. For patients with non-squamous, advanced NSCLC with PD-L1 TPS < 50% and no actionable mutations, the Task Force unanimously agreed that patients should receive first-line pembrolizumab + pemetrexed and carboplatin, based on Level A evidence (Fig. 1). For patients with non-squamous, advanced NSCLC and one or more actionable mutations (EGFR, ALK, or ROS1+), the Task Force recommends the use of targeted therapies over pembrolizumab + chemotherapy, based on Level A evidence (Fig. 1).

Concerning treatment recommendations for patients with squamous histology, the Task Force recognized that KEYNOTE-407 data has been positive thus far and FDA review is under way. As such, the Task Force decided to prospectively consider combination pembrolizumab + chemotherapy as an option for the treatment of patients with advanced, squamous cell NSCLC, and supports its use in appropriate patient subgroups if and when FDA approval is official.

In all, the majority of the Task Force recommended pembrolizumab monotherapy for patients with squamous cell NSCLC and PD-L1 TPS ≥ 50%, based on Level A evidence (Fig. 1). Some Task Force members answered that if FDA approval is granted, combination pembrolizumab + carboplatin & nab-paclitaxel/paclitaxel can be considered for this patient subgroup (Fig. 1). For patients with squamous histology and PD-L1 TPS < 50%, the Task Force unanimously recommended combination pembrolizumab + chemotherapy pending FDA approval, based on Level A evidence.

All of the above recommendations will be continually revisited and updated as new data and FDA approvals become available, especially those concerning combination nivolumab + ipilimumab, nivolumab + chemotherapy, as well as atezolizumab-based combination therapies.

#### Second-line therapy

Based on Level A evidence, the Task Force unanimously agreed that atezolizumab, nivolumab, and pembrolizumab (TPS ≥ 1%) should be used as third-line therapy in all patients with actionable mutations after disease progression with targeted agents followed by platinum-containing chemotherapy (Fig. 1). The Task Force also recommends these therapies second-line in patients with squamous histology and PD-L1 TPS < 50% who were not previously treated with a checkpoint inhibitor. In the second-line setting, the Task Force reported using pembrolizumab less frequently than nivolumab or atezolizumab in order to avoid the need for PD-L1 testing before starting treatment. However, there is no evidence to support the use of one drug over the other; this decision should depend upon patient eligibility, schedule preference (Q2W vs Q3W vs Q4W), experience of the treating physician, and drug availability/insurance coverage. In addition to PD-L1 expression (71%), the Task Force felt that history of smoking (29%) was predictive of patients who would be likely to respond to checkpoint inhibitor therapy.

#### Clinical question 2: What is the role of PD-L1 testing in determining eligibility for treatment with immune checkpoint inhibitors?

Currently, four assays that utilize distinct antibody clones (22C3, 28–8, SP263, and SP142), unique assay conditions, and independent scoring systems are FDA-approved as either complementary (to aid in patient selection) or companion (required prior to the initiation of treatment) diagnostics to measure PD-L1 expression in patients with NSCLC. Several of these are also approved in other indications. Although PD-L1 has been shown to be a predictive biomarker for response to checkpoint inhibitor therapy in NSCLC, it is neither fully sensitive nor specific. Moreover, variation in both the clinical indication and technical aspects of the standardized PD-L1 IHC assays has led to uncertainty about their optimal use (see Table 1). The Task Force, therefore, discussed the preferred tissue for PD-L1 testing (archived or fresh tissue), optimal assay and antibody, when to test and initiate treatment, and whether to retest PD-L1-negative patients after disease progression.

**Table 1:** PD-L1 assay characteristics and performance in NSCLC

Assay	Antibody	FDA-approved Indication in NSCLC	Cutoff Value	Performance^a^
22C3 IHC pharmDx	Monoclonal mouse anti-PD-L1, Clone 223	Approved as a companion diagnostic to select patients with advanced NSCLC for treatment with pembrolizumab first-line (TPS ≥ 50%) or after progression on a platinum containing chemotherapy regimen (TPS ≥ 1%)	• TPS < 1% PD-L1 negative • TPS 1–49% PD-L1 positive • TPS ≥ 50% High PD-L1 expression	Found to be closely aligned with 28–8 and SP263 IHC assays
28–8 IHC pharmDx	Monoclonal Rabbit anti-PD-L1, Clone 28–8	Approved as a complementary diagnostic to aid in non-squamous NSCLC patient selection for treatment with nivolumab	Qualitative test reported as a percentage of tumor cells exhibiting positive membrane staining	Found to be closely aligned with 22C3 and SP263 IHC assays
PD-L1 (SP142) Assay	Monoclonal Rabbit anti-PD-L1, Clone SP142	Approved as a complementary diagnostic to aid in NSCLC patient selection for treatment with atezolizumab	PD-L1 expression in ≥ 50% tumor cells or ≥ 10% immune-infiltrating cells is associated with enhanced survival	Consistently stained fewer PD-L1 tumor cells
PD-L1 (SP263) Assay	Monoclonal Rabbit anti-PD-L1, Clone SP263	CE mark only, not approved by the FDA for patients with NSCLC	The CE mark was granted and expanded based on demonstrated equivalency to the 28–8 and the 22C3 IHC assays	Found to be closely aligned with 22C3 and 28–8 IHC assays

*Abbreviations IHC* immunohistochemistry, *NSCLC* non-small cell lung cancer, *PD-L1* programmed cell death ligand 1, *TPS* tumor proportion score

^a^As assessed in Phase I of the Blueprint PD-L1 IHC assay Comparison Project [56]

### Literature review and analysis

#### PD-L1 expression analysis as a complementary diagnostic

Based on early studies showing correlation between PD-L1 expression and clinical benefit from nivolumab [21, 49], the 28–8 pharmDx test was developed as a standardized IHC assay to measure the proportion of tumor cells that express PD-L1. Whether PD-L1 expression is predictive of response to nivolumab remains unclear. In patients with squamous cell NSCLC, tumor PD-L1 expression did not correlate with clinical benefit from nivolumab [28, 50]. However, in a retrospective analysis of tumor samples from a phase III study of nivolumab vs. docetaxel in patients with NSCLC, PD-L1 expression ≥ 1, ≥ 5, and ≥ 10% was associated with longer OS and PFS with nivolumab compared to chemotherapy [29]. In these studies a small portion of patients classified as PD-L1-negative also experienced clinical benefit from nivolumab. The 28–8 assay was therefore labeled as a complementary diagnostic assay by the FDA.

The PD-L1 IHC assay, using clone SP142, was employed to determine eligibility for the randomized, phase II trials evaluating atezolizumab as first or subsequent-line therapy [42], or atezolizumab vs. docetaxel [42, 43, 51] in previously treated patients with NSCLC. In this assay, PD-L1 positivity is categorized by cell type – tumor (TC) or immune cell (IC) – and scored by the proportion of expressing cells (< 1% [TC0 or IC0], 1–4% [TC1 or IC1], 5–49% [TC2 or IC2], and ≥ 50% [TC3 or IC3]). Based on the improvement in OS associated with PD-L1 expression (TC1/2/3 or IC1/2/3) in these studies, the SP142 assay was used to stratify patients in the phase III study supporting the FDA approval of atezolizumab. Although the co-primary endpoint of the study was OS in the PD-L1-positive population (TC1/2/3 or IC1/2/3), patients with low or undetectable PD-L1 expression (TC0 or IC0) also demonstrated improved OS with atezolizumab (12.6 months vs. 8.9 months; HR 0.75, 95% CI: 0.59–0.96) [45]. Accordingly, the PD-L1 SP142 assay was labeled as a complementary diagnostic and is not required prior to initiating treatment with atezolizumab in this setting.

#### PD-L1 expression analysis as a companion diagnostic

Currently, the 22C3 pharmDx assay is the only PD-L1 assay labeled as a companion diagnostic. Its use is therefore required prior to initiating first-line treatment with pembrolizumab monotherapy, and following disease progression on platinum-based chemotherapy. A relationship between PD-L1 expression and pembrolizumab was initially observed in early phase I testing [52], resulting in an amendment to the protocol to only include patients whose tumors had a TPS ≥ 1%. A co-primary efficacy endpoint was also added in patients with tumors that expressed a high level of PD-L1, based on an optimal cutoff for PD-L1 positivity of ≥ 50% [34]. The subsequent phase II/III study of pembrolizumab vs. docetaxel for previously treated NSCLC used the 22C3 pharmDx test to classify patients into three categories based on expression of PD-L1: high (TPS ≥50%), intermediate (TPS 1–49%), or low (TPS < 1%) [35]. In patients with high PD-L1 TPS, OS was significantly longer in the 2 mg/kg pembrolizumab cohort (HR 0.54, 95% CI: 0.38–0.77, *P* = 0.0002) and the 10 mg/kg pembrolizumab cohort (HR 0.50, 95% CI: 0.36–0.70; *P* < 0.0001) compared with docetaxel. Patients with a TPS ≤ 1% were excluded from this study and the 22C3 pharmDx assay was approved by the FDA as a companion diagnostic to identify patients with a PD-L1 TPS ≥ 1% in October 2016. Based on these results, the phase III trial of pembrolizumab in untreated patients included only those with a PD-L1 TPS ≥ 50% [37]. The 22C3 pharmDx assay was therefore labeled as a companion diagnostic to identify patients eligible to receive first-line pembrolizumab (TPS ≥ 50%).

#### Laboratory-developed PD-L1 assays

In addition to the FDA-approved commercial assays, laboratory-developed tests (LDTs) have been developed in Clinical Laboratory Improvement Amendments (CLIA)-certified laboratories to measure PD-L1 expression. LDTs are tests developed, manufactured, and used within a single laboratory and are not currently required by the FDA to demonstrate clinical validity [53]; however, CLIA requires evidence of certain performance metrics to demonstrate the analytical validity of the assay. The antibodies designed to measure PD-L1 expression in LTDs have shown high concordance with FDA-approved assays, which suggests that assays using distinct antibody clones have the potential to yield concordant results if properly validated [54].One such example is an LDT that utilizes the E1L3N antibody clone to detect PD-L1 expression and has demonstrated analytical concordance with the 22C3 and 28–8 assays [55]. LTDs with analytical validity have been developed for PD-L1 and are used by many institutions to measure PD-L1 expression.

#### PD-L1 assay compatibility

The Blueprint PD-L1 IHC Assay Comparison Project was designed to compare the analytical and clinical compatibility of the available PD-L1 assays. In the first phase of this study, 39 NSCLC tumors were stained with one of the PD-L1 IHC assays (22C3, 28–8, SP142, or SP263) and evaluated for the proportion of tumor and immune cells staining positive for PD-L1 at any intensity [56]. The diagnostic performance of each assay was assessed by comparing how experts classified patients based on (above or below) a given cutoff value for PD-L1 expression. Analytical performance was comparable between the 22C3, 28–8, and SP263 assays, but the SP142 assay was found to stain fewer cells overall. However, the applicability of these results is limited as the study was underpowered and did not include an LDT [56].

The analytical performance of four PD-L1 assay platforms using the antibody clones 28–8, 22C3, SP142, and E1L3N was also compared in a prospective, multi-institutional study [55]. In this study, serial histological sections from 90 archival NSCLC tissue specimens were distributed to 3 independent sites to perform each assay. The resulting slides were scanned and scored by 13 pathologists who estimated the percentage of tumor and immune cells expressing PD-L1. Consistent with the results of the Blueprint project, the SP142 assay demonstrated a significantly lower mean PD-L1 expression score in both tumor and immune cells. However, the 28–8, 22C3, and E1L3N assays showed high concordance and the resulting classifications were found to be reproducible when read by pathologists. Subsequently, other studies carried out using LDTs developed with identical as well as distinct antibody clones have also shown analytic compatibility with FDA-approved platforms [57]. Thus, as long as the assays are carefully validated, LDTs can provide a reliable measurement of PD-L1 expression.

### Consensus recommendations

There is unanimous agreement that PD-L1 testing should be performed in newly diagnosed patients with metastatic disease, including those tested for EGFR/ALK/ROS1 mutations whose results are awaited, based on Level A evidence from multiple studies. The Task Force reported using PD-L1 testing in almost 100% of patients with newly diagnosed metastatic NSCLC. Responses varied on the use of archived or fresh biopsy tissue for PD-L1 testing: the majority of Task Force members reported using archived tissue blocks, if available, and obtaining fresh tissue as needed. Previously cut slides < 3 months old may also be used to measure PD-L1 staining. Nearly all Task Force members (83%) reported performing PD-L1 testing locally, and all reported waiting for PD-L1 test results before initiating treatment in the first-line setting. The majority (72%) of Task Force members did not retest PD-L1-negative patients after disease progression on first-line therapy.

Of note, clinical trial data concerning combination nivolumab + ipilimumab from CheckMate 227 indicate that tumor mutational burden may also be predictive of therapeutic efficacy in patients with advanced NSCLC, independent of PD-L1 status [33, 58]. As such, the Task Force recognizes that testing for tumor mutational burden may become appropriate as studies mature and new therapies are granted FDA approval.

#### Clinical question 3: How should radiographic response to immune checkpoint inhibitors be measured and monitored?

Unlike cytotoxic or targeted agents that act directly on malignant cells, immune checkpoint inhibitors enhance the immune system’s ability to recognize and eliminate cancer cells. These therapies are associated with distinct response kinetics and radiographic response patterns that make monitoring clinical response challenging. In particular, the phenomenon of pseudoprogression, defined as an initial increase in tumor burden or appearance of new lesions followed by a response to therapy, has been described in patients with NSCLC receiving immune checkpoint inhibitor therapy. To address these challenges, the Task Force discussed radiographic monitoring of clinical response in patients receiving immunotherapy.

#### Literature review and analysis

A set of immune-related response criteria (irRC) has been developed for use in clinical trials of immunotherapy [59]. The key distinguishing features of these criteria are 1) inclusion of new lesions in the total tumor burden, and 2) a requirement for confirmation of progressive disease (appearance of new lesions or tumor burden increase of > 20%) on two consecutive scans at least 4 weeks apart [59]. irRC were later optimized to increase concordance with traditional Response Evaluation Criteria in Solid Tumors v1.1 (RECIST1.1), and there is now the option to use either traditional or immune-related RECIST response criteria when evaluating treatment response in cancer immunotherapy trials [60, 61]. However, since the majority of clinical trials that led to FDA approval of checkpoint inhibitors predate the introduction of iRC, data from these trials may fail to capture unique immune-related response patterns, such as pseudoprogression [62].

Two small retrospective studies compared RECIST1.1 with irRC to identify patients with NSCLC who were wrongly classified in clinical trials as having progressive disease [63, 64]. In these studies, pseudoprogression was very infrequently observed in patients with NSCLC, with the highest frequency reported being 2/41 (4.9%) patients [63]. Although pseudoprogression was particularly seen in patients with melanoma, it seems to be very uncommon in NSCLC. Further study is warranted to determine whether treatment with immune checkpoint inhibitors beyond RECIST1.1 disease progression benefits patients with NSCLC.

#### Consensus recommendations

In the absence of robust data, the majority of Task Force members (62%) reported obtaining the first CT scan 6–9 weeks after starting immune checkpoint inhibitor therapy. If asymptomatic or minimal disease progression is observed at this time, most Task Force members (69%) would continue treatment as long as the patient was clinically stable. In cases where treatment with an immune checkpoint inhibitor is continued beyond evidence of disease progression, the majority of Task Force members would repeat a CT scan after 4 weeks (31%) or after 8 weeks (39%).

#### Clinical question 4: Should patients with NSCLC and a co-existing autoimmune disorder be treated with immune checkpoint inhibitors?

Immune checkpoint inhibitors are often withheld from patients with preexisting or active autoimmune disorders based on the assumption that autoimmune toxicity could be exacerbated. However, because individuals with active autoimmune disease have typically been excluded from clinical trials of immunotherapy, data are insufficient to determine whether immune-based therapies are contraindicated. The Task Force, therefore, discussed whether autoimmune disorders are a contraindication to treatment, including whether the type and/or severity of autoimmune disease could affect patient eligibility.

#### Literature review and analysis

There are limited data concerning the use of any checkpoint inhibitors in patients with preexisting autoimmune disorders. In a retrospective review of patients with advanced melanoma who received ipilimumab therapy, 30 patients in the treatment arm had active preexisting autoimmune disorders (rheumatoid arthritis, *n* = 6; psoriasis, *n* = 5; inflammatory bowel disease, *n* = 6; systemic lupus erythematosus, *n* = 2; multiple sclerosis *n* = 2; autoimmune thyroiditis, *n* = 2; other, *n* = 7), and 43% (13/30) were receiving immunosuppressive therapy [65]. Following ipilimumab treatment, 8 patients (27%) required corticosteroid treatment for exacerbation of an autoimmune condition. Severe (grade 3–5) irAEs occurred in 10 patients, of whom 2 responded fully to corticosteroids or infliximab; one patient (psoriasis) died of presumed immune-related colitis. Fifteen patients had no autoimmune disease flare. In all, 6 patients (20%) experienced an objective response, with a single durable complete response [65].

The same clinical question was addressed in a systematic review of 45 cases, the majority of which involved patients with melanoma and an autoimmune disorder (95.6%) who received ipilimumab (88.9%). In this review, 40% of patients did not experience irAEs or disease worsening despite having active autoimmune disease at the time of treatment [66].

In a small prospective study, 119 patients with advanced melanoma, 52 with preexisting autoimmune disorders and 67 with major toxicity with ipilimumab, were treated with anti-PD-1 agents (109 pembrolizumab and 10 nivolumab). Among patients with preexisting autoimmune disorders, the response rate was 33%. Twenty (38%) patients reported autoimmune flares requiring immunosuppression, including 7/13 with rheumatoid arthritis, 3/3 with polymyalgia rheumatica, 2/2 with Sjögren’s syndrome, 2/2 with immune thrombocytopenic purpura, and 3/8 with psoriasis; only 2 (4%) patients discontinued treatment due to autoimmune flare, and there were no treatment-related deaths [67].

The literature on immunotherapy in organ transplant recipients is extremely limited. A systematic review identified 19 cases of cancer patients who had received solid organ transplant (Cancer type: melanoma = 11, cutaneous squamous cell = 3, NSCLC = 2, hepatocellular = 2, duodenal = 1; transplant type: kidney = 12, liver = 5, heart = 2) being treated with checkpoint inhibitors (median time to start therapy: 11 years; 53% nivolumab, 26% ipilimumab, 21% pembrolizumab). Most patients were receiving immunosuppressive regimens – including low-dose prednisone and mTOR inhibitors – prior to initiating checkpoint inhibitor therapy. Ten patients experienced graft rejection (7 kidney, 2 liver, 1 heart) after checkpoint inhibitor therapy (median time to rejection = 21 days). Biopsy samples suggested T cell mediated rejection [68]. There are currently no guidelines on the use of immunotherapy in transplant recipients, and more research is needed to clarify the safety and efficacy of immunotherapy in this setting.

Although there may be increased risk of toxicity in patients with autoimmune conditions, and among those with an organ transplant, published reports indicate that toxicity is not universal and benefits can be seen.

#### Consensus recommendations

The Task Force recognized that very little is known about contraindications to immunotherapy in patients with NSCLC, and that many of the above examples concern anti-CTLA-4 ipilimumab that hold no approvals for this disease. Because patients with autoimmune disease are typically excluded from immunotherapy clinical trials, the use of checkpoint inhibitors in these patients is still considered investigational. Only 6% of the Task Force felt that a history of multiple sclerosis would be an absolute contraindication. Furthermore, in the context of an otherwise fatal illness such as lung cancer there may be greater willingness to accept the risk of toxicity, particularly in the absence of alternative effective therapies. Of note, the majority of the Task Force (75%) felt that prior liver transplant was an absolute contraindication to immune checkpoint therapy as some deaths and organ rejection have been described. Until further data are available, particularly from real-world clinical settings, close monitoring in conjunction with appropriate specialist care is recommended to ensure early identification and effective management of irAEs.

#### Clinical question 5: How should treatment-related adverse events, in particular pulmonary adverse events, be recognized, monitored, and managed in patients with NSCLC?

Cancer immunotherapy agents are associated with toxicities that are distinct from those observed with cytotoxic or targeted agents. Early recognition and close monitoring of these toxicities can improve clinical outcomes while minimizing harm to patients. Overall, serious immune-related toxicities are quite rare. Treatment-related pneumonitis has been reported as a cause of death in patients with NSCLC, but this occurs in < 2% of patients [69]. The overall incidence of individual immune-related toxicities is low, but the absolute burden on patients is substantial due to the broad use of these agents. The prevalence of irAEs may also increase with future use of combination regimens.

#### Literature review and analysis

Data concerning the incidence of pulmonary irAEs have been mostly reported in large prospective trials supporting FDA-approval of the agents in question. According to these safety trials, immune-mediated pneumonitis was observed in 61/1994 (3.1%) of patients receiving nivolumab, 94/2799 (3.4%) of patients receiving pembrolizumab, and 38/1027 (3.7%) of patients who received atezolizumab [44]. The median time to onset of immune-mediated pneumonitis was 3.5 months for nivolumab and 3.3 months for both pembrolizumab and atezolizumab. The majority of patients who developed pneumonitis while undergoing treatment were managed with corticosteroids (89, 67, and 55%, respectively). Although most irAEs were grade 1–2 and eventually resolved, two immune-related pneumonitis deaths were reported.

Consistent with safety reports, a large retrospective analysis of patients receiving anti-PD-1/PD-L1 agents reported immune-related pneumonitis in 43/915 (4.6%) patients [70], with similar incidence in patients with NSCLC (26/532 [5%]) and melanoma (9/209 [4%]). Time to onset ranged from 9 days to 19.2 months (median 2.8 months) and was shorter in patients treated with combination therapy compared with single agents (median 2.7 vs 4.6 months; *p* = 0.02). Of the reported cases, 72% were grade 1 to 2, and 86% improved or resolved once immunotherapy was withheld and immunosuppression initiated. Treatment for pneumonitis included withholding drug (*n* = 15, all grade 1), initiating corticosteroids (*n* = 23, 2 grade 1, 14 grade 2, 6 grade 3, 1 grade 4), and using corticosteroids with additional immunosuppression from infliximab with or without cyclophosphamide (*n* = 5, all grade ≥ 3). During treatment for pneumonitis, five patients died but only one death was directly attributable to pneumonitis. Of note, three patients died from infections related to immunosuppression, highlighting the need for improved immunosuppression strategies [70].

Risk of pneumonitis is generally increased in patients with NSCLC, including after radiation and chemotherapy [71–73]. Concerning pneumonitis caused by checkpoint inhibition, a meta-analysis of 4496 patients across 20 PD-1/PD-L1 trials, the frequency of pneumonitis was found to be higher in patients treated with combination compared with monotherapy regimens (all-grade: 6.6% vs. 1.6%; *p* < 0.001; grade ≥ 3: 1.5% vs. 0.2%, *p* = 0.001) [74]. In addition, patients with NSCLC were found to have a higher incidence of all-grade (4.1% vs 1.6%; *p* = 0.002) and grade ≥ 3 pneumonitis (1.8% vs. 0.2%; *p* < 0 .001) compared with patients with melanoma. Moreover, in a large meta-analysis of more than 5000 NSCLC patients, the incidence of any-grade (3.6% vs. 1.3%) and grade ≥ 3/4 (1.1% vs. 0.4%) pneumonitis was higher with PD-1 inhibitors than PD-L1 inhibitors [75]. Treatment-naïve patients were found to have a higher incidence of grade 1–4 pneumonitis compared with previously treated patients (4.3% vs. 2.8%) [75]. Predictors of immune-related toxicity remain to be clarified although family history of autoimmune disease, concomitant use of other agents with known autoimmune effects, tumor location, previous viral infection and elevated serum levels of eosinophils and IL-17 have all been proposed based on varying degrees of evidence [76]. It is important to note that irAEs can present after cessation of immunotherapy. For example, patients with breast cancer enrolled in KEYNOTE 012 developed irAEs over a year after halting pembrolizumab [77].

#### Consensus recommendations

In order to ensure irAEs are properly identified and managed, the Task Force recommended close monitoring and cross-collaboration with disease specialists. When managing immune-related toxicities, over 50% of the Task Force routinely collaborated with radiologists (79%), pulmonologists (71%), dermatologists (71%), rheumatologists (71%), and endocrinologists (71%). In addition to the baseline tests recommended prior to starting immunotherapy (described earlier), tests routinely used by ≥50% of Task Force members to monitor patients treated with immune checkpoint inhibitors included: thyroid function studies (93%), liver function tests (93%), blood urea nitrogen (BUN) and creatinine (86%), and whole body imaging (71%). The importance of closely monitoring patients’ oxygen saturation at rest and on ambulation was also noted.

To ensure prompt diagnosis and management of pneumonitis, the Task Force recommended frequent monitoring of, and patient education on, signs or symptoms of possible pneumonitis such as new or worsening cough, wheezing, dyspnea, or fatigue. In addition, all patients with radiographic and/or clinical evidence of pneumonitis should be referred to a pulmonary specialist. In cases of grade 2 pneumonitis, immunotherapy should be withheld and steroids (e.g., prednisone 1 mg/kg daily) administered. Grade 3/4 pneumonitis warrants permanently discontinuing immunotherapy and initiating treatment with steroids, including consideration of IV steroids and hospitalization. Specific recommendations on the management of pulmonary irAEs are provided in detail by SITC’s Toxicity Management Working Group [78].

## Conclusions

With encouraging clinical activity, manageable side effects, and the potential for durable responses, immune checkpoint inhibitors have quickly become the standard of care for eligible patients with NSCLC within academic centers. Currently, clinical trials with cancer immunotherapy agents alone and in combination with other immune-based agents, targeted therapies, and cytotoxic agents (chemotherapy and radiation therapy) are underway [79]. The eagerly anticipated results from these trials will determine what role these agents will play in treating patients with early stage disease, including the neoadjuvant and adjuvant settings. In addition to advances in treatment strategies, identifying and refining the use of predictive biomarkers will also be essential to identify patients who will most likely benefit from therapy. Practice-changing updates from ongoing studies will be incorporated into future versions of this guideline document.

## Additional files

Additional file 1: Cancer Immunotherapy Guidelines- Lung Task Force Roster. (DOCX 13 kb)

Additional file 2: Comments from Member Open Review. (DOCX 14 kb)

Additional file 3: SITC NSCLC CIG Bibliography. (DOCX 62 kb)

## Abbreviations

ALK: Anaplastic lymphoma kinase
CT: Computerized tomography
EGFR: Epidermal growth factor receptor
FDA: U.S. Food and Drug Administration
IC: Immune cell
IHC: Immunohistochemistry
irAEs: Immune-related adverse events
irRC: Immune-related response criteria
MMF: Mycophenolate mofetil
MRI: Magnetic resonance imaging
NSCLC: Non-small cell lung cancer
ORR: Overall response rate
OS: Overall survival
PD-1: Programmed cell death 1
PD-L1: Programmed cell death ligand 1
PFS: Progression-free survival
RECIST1.1: Response Evaluation Criteria in Solid Tumors v1.1
SITC: Society for Immunotherapy of Cancer
TC: Tumor cell
TPS: Tumor proportion score
