# Supplementary material for: The Society for Immunotherapy of Cancer consensus statement on immunotherapy for the treatment of non-small cell lung cancer (NSCLC)
Source: J Immunother Cancer. 2018 Jul 17;6:75. doi: 10.1186/s40425-018-0382-2 (PMC6048854; doi:10.1186/s40425-018-0382-2)
Supplement: Supplementary file 1 — Cancer Immunotherapy Guidelines- Lung Task Force Roster. (DOCX 13 kb) [file 40425_2018_382_MOESM1_ESM.docx]

**ADDITIONAL FILE I:**

**Cancer Immunotherapy Guidelines- Lung Task Force Roster**

**Steering Committee:**

Roy S. Herbst, MD, PhD, Yale Cancer Center

Julie R. Brahmer, MD, Sidney Kimmel Comprehensive Cancer Center at Johns Hopkins

Ramaswamy Govindan, MD, Washington University School of Medicine

Naiyer A. Rizvi, MD, Columbia University Medical Center

**Task Force Participants:**

Robert A. Anders, MD, PhD, Johns Hopkins School of Medicine

Scott J. Antonia, MD, PhD, H. Lee Moffitt Cancer Center and Research Institute

Sarah Sagorsky, PA, Johns Hopkins Kimmel Cancer Center

Marianne J. Davies, DNP, AOCNP, Yale Comprehensive Cancer Center

Steven M. Dubinett, MD, University of California Los Angeles Lung Cancer Research Program

Andrea Ferris, LUNGevity

Leena Gandhi, MD, PhD, New York University

Edward B. Garon, MD, University of California Los Angeles

Matthew D. Hellmann, MD, Memorial Sloan Kettering Cancer Center

Fred R. Hirsch, MD, PhD, University of Colorado Denver School of Medicine

Shakuntala Malik, MD, National Cancer Institute

Joel W. Neal, MD, PhD, Stanford University School of Medicine

Vassiliki A. Papadimitrakopoulou, MD, University of Texas MD Anderson Cancer Center

David L. Rimm, MD, PhD, Yale University School of Medicine

Lawrence H. Schwartz, MD, Columbia University College of Physicians and Surgeons

Boris Sepesi, MD, University of Texas MD Anderson Cancer Center

Beow Yong Yeap, Massachusetts General Hospital
