# Supplementary material for: The Society for Immunotherapy of Cancer consensus statement on immunotherapy for the treatment of non-small cell lung cancer (NSCLC)
Source: J Immunother Cancer. 2018 Jul 17;6:75. doi: 10.1186/s40425-018-0382-2 (PMC6048854; doi:10.1186/s40425-018-0382-2)
Supplement: Supplementary file 2 — Comments from Member Open Review. (DOCX 14 kb) [file 40425_2018_382_MOESM2_ESM.docx]

**ADDITIONAL FILE 2: Comments from Member Open Review**

Comments from the member open review of this consensus statement will be published with this document.

| **Comment Date** | **Comments** |
| --- | --- |
| **4/18/2018** | Correction starting at line 308. Most recently, a phase III trial comparing first line pembrolizumab plus chemotherapy (pemetrexed plus cisplatin or carboplatin) versus chemotherapy alone in 616 patients with advanced or metastatic NSCLC, irrespective of PD-L1 expression, met its co-primary endpoints of improved OS and PFS. |
| **4/18/2018** | - Line 79, 216, 355-356: Suggest adding the word "unresectable stage III" to describe durvalumab indication - Line 235: Nivolumab is listed as a "fully humanized" mAb, whereas it is actually a fully human mAb. Pembrolizumab is correctly stated as "fully humanized" (line 255) - Line 244-245: The way this is written suggests that nivolumab was approved at the 240 mg flat dose. - Line 247: Suggest adding that long-term 3-year data from these studies demonstrate durable responses and OS (Annals of Oncology) - Line 248-250: The 541 patients included in CM026 were at >1% PD-L1 expression but line 250 suggests they were at >5%, which was the primary endpoint population. Only 423 patients had >5% PD-L1 expression. - Line 271-272: The way this is written suggests that pembrolizumab was approved at the 200 mg flat dose. - Line 287-288: Should specify the KN021G was in nonsquamous only. - Line 383: It states "no evidence to use one drug over the other" but this does omit the fact that pembrolizumab is not approved for 2L PD-L1 non-expressors. - Line 384 (and throughout): Can mention of the nivolumab 480 mg Q4W dose and schedule be added wherever relevant? |
