# Supplementary material for: The Society for Immunotherapy of Cancer consensus statement on immunotherapy for the treatment of non-small cell lung cancer (NSCLC)
Source: J Immunother Cancer. 2018 Jul 17;6:75. doi: 10.1186/s40425-018-0382-2 (PMC6048854; doi:10.1186/s40425-018-0382-2)
Supplement: Supplementary file 3 — SITC NSCLC CIG Bibliography. (DOCX 62 kb) [file 40425_2018_382_MOESM3_ESM.docx]

**CANCER IMMUNOTHERAPY**

**GUIDELINES:**

**NON-SMALL CELL**

**LUNG CANCER**

**A bibliography of the literature**

**SOCIETY FOR IMMUNOTHERAPY OF CANCER February 14^th^, 2018**

**Table of Contents**

[TOPIC: Non-Small Cell Lung Cancer + Nivolumab 6](#_Toc161730)

[TOPIC: Non-Small Cell Lung Cancer + Ipilimumab 9](#_Toc161731)

[TOPIC: Non-Small Cell Lung Cancer + Pembrolizumab 9](#_Toc161732)

[TOPIC: Non-Small Cell Lung Cancer + Durvalumab 11](#_Toc161733)

[TOPIC: Non-Small Cell Lung Cancer + Atezolizumab 12](#_Toc161734)

[TOPIC: Non-Small Cell Lung Cancer + Vaccine 12](#_Toc161734)

[TOPIC: Non-Small Cell Lung Cancer + Immunotherapy 19](#_Toc161737)

[TOPIC: Non-Small Cell Lung Cancer + PD-1/PD-L1 25](#_Toc161738)

[TOPIC: Non-Small Cell Lung Cancer + Combination Immunotherapy 26](#_Toc161738)

[TOPIC: Non-Small Cell Lung Cancer + Immunotherapy Adverse Events 27](#_Toc161739)

| **Search Terms** | **Date Limits** | **Limits** | **Search Details** | **Total Refs Identified** | **Total # Removed (duplicates)** | **Resulting # of Records in Bibliography** | **Record Numbers** |
| --- | --- | --- | --- | --- | --- | --- | --- |
| All terms | 1/1/2008-  2/10/2018 | Clinical Trial, Randomized Controlled Trial, Meta-Analysis, Practice Guideline |  | 269 | 118 | 151 | 1 - 151 |
| Non-Small Cell Lung Cancer + nivolumab | 1/1/2008-  2/10/2018 | Clinical Trial, Randomized Controlled Trial, Meta-Analysis, Practice Guideline | "Carcinoma, Non-Small-Cell Lung"[Mesh] AND "nivolumab"[Supplementary Concept] AND ((Clinical Study[ptyp] OR Clinical Trial[ptyp] OR Controlled Clinical Trial[ptyp] OR Meta-Analysis[ptyp] OR Practice Guideline[ptyp] OR Randomized Controlled Trial[ptyp]) AND ("2008/01/01"[PDAT] : "2018/02/10"[PDAT])) | 27 | 6 | 21 | 1 - 21 |
| Non-Small Cell Lung Cancer + ipilimumab | 1/1/2008-  2/10/2018 | Clinical Trial, Randomized Controlled Trial, Meta-Analysis, Practice Guideline | “Carcinoma, Non-Small-Cell Lung"[Mesh] AND "ipilimumab"[Supplementary Concept] AND ((Clinical Study[ptyp] OR Clinical Trial[ptyp] OR Controlled Clinical Trial[ptyp] OR Meta-Analysis[ptyp] OR Practice Guideline[ptyp] OR Randomized Controlled Trial[ptyp]) AND ("2008/01/01"[PDAT] : "2018/02/10"[PDAT])) | 6 | 2 | 4 | 22 - 25 |
| Non-Small Cell Lung Cancer + pembrolizumab | 1/1/2008-  2/10/2018 | Clinical Trial, Randomized Controlled Trial, Meta-Analysis, Practice Guideline | “Carcinoma, Non-Small-Cell Lung"[Mesh] AND "pembrolizumab"[Supplementary Concept] AND ((Clinical Study[ptyp] OR Clinical Trial[ptyp] OR Controlled Clinical Trial[ptyp] OR Meta-Analysis[ptyp] OR Practice Guideline[ptyp] OR Randomized Controlled Trial[ptyp]) AND ("2008/01/01"[PDAT] : "2018/02/10"[PDAT])) | 19 | 6 | 13 | 26 - 38 |
| Non-Small Cell Lung Cancer + durvalumab | 1/1/2008-  2/10/2018 | Clinical Trial, Randomized Controlled Trial, Meta-Analysis, Practice Guideline | “Carcinoma, Non-Small-Cell Lung"[Mesh] AND ("durvalumab"[Supplementary Concept] OR "MEDI4736" [Supplementary Concept]) AND ((Clinical Study[ptyp] OR Clinical Trial[ptyp] OR Controlled Clinical Trial[ptyp] OR Meta-Analysis[ptyp] OR Practice Guideline[ptyp] OR Randomized Controlled Trial[ptyp]) AND ("2008/01/01"[PDAT] : "2018/02/10"[PDAT])) | 5 | 1 | 4 | 39 - 42 |
| Non-Small Cell Lung Cancer + atezolizumab | 1/1/2008-  2/10/2018 | Clinical Trial, Randomized Controlled Trial, Meta-Analysis, Practice Guideline | "Carcinoma, Non-Small-Cell Lung"[Mesh] AND "atezolizumab"[Supplementary Concept] AND ((Clinical Study[ptyp] OR Clinical Trial[ptyp] OR Controlled Clinical Trial[ptyp] OR Meta-Analysis[ptyp] OR Practice Guideline[ptyp] OR Randomized Controlled Trial[ptyp]) AND ("2008/01/01"[PDAT] : "2018/02/10"[PDAT])) | 6 | 4 | 2 | 43 - 44 |
| Non-Small Cell Lung Cancer + vaccine | 1/1/2008-  2/10/2018 | Clinical Trial, Randomized Controlled Trial, Meta-Analysis, Practice Guideline | “Carcinoma, Non-Small-Cell Lung"[Mesh] AND "Vaccines"[Mesh] AND ((Clinical Study[ptyp] OR Clinical Trial[ptyp] OR Controlled Clinical Trial[ptyp] OR Meta-Analysis[ptyp] OR Practice Guideline[ptyp] OR Randomized Controlled Trial[ptyp]) AND ("2008/01/01"[PDAT] : "2018/02/10"[PDAT])) | 47 | 1 | 46 | 45 - 90 |
| Non-Small Cell Lung Cancer + immunotherapy | 1/1/2008-  2/10/2018 | Clinical Trial, Randomized Controlled Trial, Meta-Analysis, Practice Guideline | “Carcinoma, Non-Small-Cell Lung"[Mesh] AND "Immunotherapy"[Mesh] AND ((Clinical Study[ptyp] OR Clinical Trial[ptyp] OR Controlled Clinical Trial[ptyp] OR Meta-Analysis[ptyp] OR Practice Guideline[ptyp] OR Randomized Controlled Trial[ptyp]) AND ("2008/01/01"[PDAT] : "2018/02/10"[PDAT])) | 67 | 23 | 44 | 91 - 134 |
| Non-Small Cell Lung Cancer + PD-1/PD-L1 | 1/1/2008-  2/10/2018 | Clinical Trial, Randomized Controlled Trial, Meta-Analysis, Practice Guideline | "Carcinoma, Non-Small-Cell Lung "[Mesh] AND (PD-1[All fields] OR ("antigens, cd274"[MeSH Terms] OR ("antigens"[All Fields] AND "cd274"[All Fields]) OR "cd274 antigens"[All Fields] OR ("pd"[All Fields] AND "l1"[All Fields]) OR "pd l1"[All Fields])) AND ((Clinical Study[ptyp] OR Clinical Trial[ptyp] OR Controlled Clinical Trial[ptyp] OR Meta-Analysis[ptyp] OR Practice Guideline[ptyp] OR Randomized Controlled Trial[ptyp]) AND ("2008/01/01"[PDAT] : "2018/02/10"[PDAT])) | 50 | 37 | 13 | 135 - 147 |
| Non-Small Cell Lung Cancer + combination immunotherapy | 1/1/2008-  2/10/2018 | Clinical Trial, Randomized Controlled Trial, Meta-Analysis, Practice Guideline | “Carcinoma, Non-Small-Cell Lung"[Mesh] AND "Drug Therapy, Combination"[Mesh] AND "Immunotherapy"[Mesh] AND ((Clinical Study[ptyp] OR Clinical Trial[ptyp] OR Controlled Clinical Trial[ptyp] OR Meta-Analysis[ptyp] OR Practice Guideline[ptyp] OR Randomized Controlled Trial[ptyp]) AND ("2008/01/01"[PDAT] : "2018/02/10"[PDAT])) | 17 | 17 | 0 | N/A |
| Non-Small Cell Lung Cancer + Immunotherapy Adverse Events | 1/1/2008-  2/10/2018 | Clinical Trial, Randomized Controlled Trial, Meta-Analysis, Practice Guideline | “Carcinoma, Non-Small-Cell Lung"[Mesh]) AND (("adverse events"[All Fields] OR "side effects"[All Fields]) AND ("immunotherapy"[Mesh]) AND ((Clinical Study[ptyp] OR Clinical Trial[ptyp] OR Controlled Clinical Trial[ptyp] OR Meta-Analysis[ptyp] OR Practice Guideline[ptyp] OR Randomized Controlled Trial[ptyp]) AND ("2008/01/01"[PDAT] : "2018/02/10"[PDAT])) | 25 | 21 | 4 | 148 - 151 |

# TOPIC: Non-Small Cell Lung Cancer + Nivolumab

1. Aguiar PN, Jr., Santoro IL, Tadokoro H, de Lima Lopes G, Filardi BA, Oliveira P, Castelo-Branco P, Mountzios G, de Mello RA: **A pooled analysis of nivolumab for the treatment of advanced non-small-cell lung cancer and the role of PD-L1 as a predictive biomarker**. *Immunotherapy* 2016, **8**(9):1011-1019.
2. Borghaei H, Paz-Ares L, Horn L, Spigel DR, Steins M, Ready NE, Chow LQ, Vokes EE, Felip E, Holgado E *et al*: **Nivolumab versus Docetaxel in Advanced Nonsquamous Non-Small-Cell Lung Cancer**. *The New England journal of medicine* 2015, **373**(17):1627-1639.
3. Brahmer J, Reckamp KL, Baas P, Crino L, Eberhardt WE, Poddubskaya E, Antonia S, Pluzanski A, Vokes EE, Holgado E *et al*: **Nivolumab versus Docetaxel in Advanced Squamous-Cell Non-Small-Cell Lung Cancer**. *The New England journal of medicine* 2015, **373**(2):123-135.
4. Brahmer JR, Drake CG, Wollner I, Powderly JD, Picus J, Sharfman WH, Stankevich E, Pons A, Salay TM, McMiller TL *et al*: **Phase I study of single-agent anti-programmed death-1 (MDX-1106) in refractory solid tumors: safety, clinical activity, pharmacodynamics, and immunologic correlates**. *Journal of clinical oncology : official journal of the American Society of Clinical Oncology* 2010, **28**(19):3167-3175.
5. Brahmer JR, Tykodi SS, Chow LQ, Hwu WJ, Topalian SL, Hwu P, Drake CG, Camacho LH, Kauh J, Odunsi K *et al*: **Safety and activity of anti-PD-L1 antibody in patients with advanced cancer**. *The New England journal of medicine* 2012, **366**(26):2455-2465.
6. Brustugun OT, Sprauten M, Helland A: **Real-world data on nivolumab treatment of non-small cell lung cancer**. *Acta oncologica (Stockholm, Sweden)* 2017, **56**(3):438-440.
7. Caponnetto S, Iannantuono GM, Barchiesi G, Magri V, Gelibter A, Cortesi E: **Prolactin as a Potential Early Predictive Factor in Metastatic Non-Small Cell Lung Cancer Patients Treated with Nivolumab**. *Oncology* 2017, **93**(1):62-66.
8. Carbognin L, Pilotto S, Milella M, Vaccaro V, Brunelli M, Calio A, Cuppone F, Sperduti I, Giannarelli D, Chilosi M *et al*: **Differential Activity of Nivolumab, Pembrolizumab and MPDL3280A according to the Tumor Expression of Programmed Death-Ligand-1 (PD-L1): Sensitivity Analysis of Trials in Melanoma, Lung and Genitourinary Cancers**. *PloS one* 2015, **10**(6):e0130142.
9. Gettinger S, Rizvi NA, Chow LQ, Borghaei H, Brahmer J, Ready N, Gerber DE, Shepherd FA, Antonia S, Goldman JW *et al*: **Nivolumab Monotherapy for First-Line Treatment of Advanced Non-Small-Cell Lung Cancer**. *Journal of clinical oncology : official journal of the American Society of Clinical Oncology* 2016, **34**(25):2980-2987.
10. Gettinger SN, Horn L, Gandhi L, Spigel DR, Antonia SJ, Rizvi NA, Powderly JD, Heist RS, Carvajal RD, Jackman DM *et al*: **Overall Survival and Long-Term Safety of Nivolumab (Anti-Programmed Death 1 Antibody, BMS-936558, ONO-4538) in Patients With Previously Treated Advanced Non-Small-Cell Lung Cancer**. *Journal of clinical oncology : official journal of the American Society of Clinical Oncology* 2015, **33**(18):2004-2012.
11. Hida T, Nishio M, Nogami N, Ohe Y, Nokihara H, Sakai H, Satouchi M, Nakagawa K, Takenoyama M, Isobe H *et al*: **Efficacy and safety of nivolumab in Japanese patients with advanced or recurrent squamous non-small cell lung cancer**. *Cancer science* 2017, **108**(5):1000-1006.
12. Horn L, Spigel DR, Vokes EE, Holgado E, Ready N, Steins M, Poddubskaya E, Borghaei H, Felip E, Paz-Ares L *et al*: **Nivolumab Versus Docetaxel in Previously Treated Patients With Advanced Non-Small-Cell Lung Cancer: Two-Year Outcomes From Two Randomized, Open-Label, Phase III Trials (CheckMate 017 and CheckMate 057)**. *Journal of clinical oncology : official journal of the American Society of Clinical Oncology* 2017, **35**(35):3924-3933.
13. Kanda S, Goto K, Shiraishi H, Kubo E, Tanaka A, Utsumi H, Sunami K, Kitazono S, Mizugaki H, Horinouchi H *et al*: **Safety and efficacy of nivolumab and standard chemotherapy drug combination in patients with advanced non-small-cell lung cancer: a four arms phase Ib study**. *Annals of oncology : official journal of the European Society for Medical Oncology* 2016, **27**(12):2242-2250.
14. Kazandjian D, Khozin S, Blumenthal G, Zhang L, Tang S, Libeg M, Kluetz P, Sridhara R, Keegan P, Pazdur R: **Benefit-Risk Summary of Nivolumab for Patients With Metastatic Squamous Cell Lung Cancer After Platinum-Based Chemotherapy: A Report From the US Food and Drug Administration**. *JAMA oncology* 2016, **2**(1):118-122.
15. Kazandjian D, Suzman DL, Blumenthal G, Mushti S, He K, Libeg M, Keegan P, Pazdur R: **FDA Approval Summary: Nivolumab for the Treatment of Metastatic Non-Small Cell Lung Cancer With Progression On or After Platinum-Based Chemotherapy**. *The oncologist* 2016, **21**(5):634-642.
16. Kobayashi H, Omori S, Nakashima K, Wakuda K, Ono A, Kenmotsu H, Naito T, Murakami H, Endo M, Takahashi T: **Response to the treatment immediately before nivolumab monotherapy may predict clinical response to nivolumab in patients with non-small cell lung cancer**. *International journal of clinical oncology* 2017, **22**(4):690-697.
17. Rizvi NA, Hellmann MD, Brahmer JR, Juergens RA, Borghaei H, Gettinger S, Chow LQ, Gerber DE, Laurie SA, Goldman JW *et al*: **Nivolumab in Combination With Platinum-Based Doublet Chemotherapy for First-Line Treatment of Advanced Non-Small-Cell Lung Cancer**. *Journal of clinical oncology : official journal of the American Society of Clinical Oncology* 2016, **34**(25):2969-2979.
18. Rizvi NA, Mazieres J, Planchard D, Stinchcombe TE, Dy GK, Antonia SJ, Horn L, Lena H, Minenza E, Mennecier B *et al*: **Activity and safety of nivolumab, an anti-PD-1 immune checkpoint inhibitor, for patients with advanced, refractory squamous non-small-cell lung cancer (CheckMate 063): a phase 2, single-arm trial**. *The Lancet Oncology* 2015, **16**(3):257-265.
19. Topalian SL, Hodi FS, Brahmer JR, Gettinger SN, Smith DC, McDermott DF, Powderly JD, Carvajal RD, Sosman JA, Atkins MB *et al*: **Safety, activity, and immune correlates of anti-PD-1 antibody in cancer**. *The New England journal of medicine* 2012, **366**(26):2443-2454.
20. Wang C, Yu X, Wang W: **A meta-analysis of efficacy and safety of antibodies targeting PD-1/PD-L1 in treatment of advanced nonsmall cell lung cancer**. *Medicine* 2016, **95**(52):e5539.
21. Yaqub F: **Nivolumab for squamous-cell non-small-cell lung cancer**. *The Lancet Oncology* 2015, **16**(7):e319.

# TOPIC: Non-Small Cell Lung Cancer + Ipilimumab

1. Govindan R, Szczesna A, Ahn MJ, Schneider CP, Gonzalez Mella PF, Barlesi F, Han B, Ganea DE, Von Pawel J, Vladimirov V *et al*: **Phase III Trial of Ipilimumab Combined With Paclitaxel and Carboplatin in Advanced Squamous Non-Small-Cell Lung Cancer**. *Journal of clinical oncology : official journal of the American Society of Clinical Oncology* 2017, **35**(30):3449-3457.
2. Hellmann MD, Rizvi NA, Goldman JW, Gettinger SN, Borghaei H, Brahmer JR, Ready NE, Gerber DE, Chow LQ, Juergens RA *et al*: **Nivolumab plus ipilimumab as first-line treatment for advanced non-small-cell lung cancer (CheckMate 012): results of an open-label, phase 1, multicohort study**. *The Lancet Oncology* 2017, **18**(1):31-41.
3. Horinouchi H, Yamamoto N, Fujiwara Y, Sekine I, Nokihara H, Kubota K, Kanda S, Yagishita S, Wakui H, Kitazono S *et al*: **Phase I study of ipilimumab in phased combination with paclitaxel and carboplatin in Japanese patients with non-small-cell lung cancer**. *Investigational new drugs* 2015, **33**(4):881-889.
4. Lynch TJ, Bondarenko I, Luft A, Serwatowski P, Barlesi F, Chacko R, Sebastian M, Neal J, Lu H, Cuillerot JM *et al*: **Ipilimumab in combination with paclitaxel and carboplatin as first-line treatment in stage IIIB/IV non-small-cell lung cancer: results from a randomized, double-blind, multicenter phase II study**. *Journal of clinical oncology : official journal of the American Society of Clinical Oncology* 2012, **30**(17):2046-2054.

# TOPIC: Non-Small Cell Lung Cancer + Pembrolizumab

1. Abdel-Rahman O: **Evaluation of efficacy and safety of different pembrolizumab dose/schedules in treatment of non-small-cell lung cancer and melanoma: a systematic review**. *Immunotherapy* 2016, **8**(12):1383-1391.
2. Ayers M, Lunceford J, Nebozhyn M, Murphy E, Loboda A, Kaufman DR, Albright A, Cheng JD, Kang SP, Shankaran V *et al*: **IFN-gamma-related mRNA profile predicts clinical response to PD-1 blockade**. *The Journal of clinical investigation* 2017, **127**(8):2930-2940.
3. Brahmer JR, Rodriguez-Abreu D, Robinson AG, Hui R, Csoszi T, Fulop A, Gottfried M, Peled N, Tafreshi A, Cuffe S *et al*: **Health-related quality-of-life results for pembrolizumab versus chemotherapy in advanced, PD-L1-positive NSCLC (KEYNOTE-024): a multicentre, international, randomised, open-label phase 3 trial**. *The Lancet Oncology* 2017, **18**(12):1600-1609.
4. Chatterjee M, Turner DC, Felip E, Lena H, Cappuzzo F, Horn L, Garon EB, Hui R, Arkenau HT, Gubens MA *et al*: **Systematic evaluation of pembrolizumab dosing in patients with advanced non-small-cell lung cancer**. *Annals of oncology : official journal of the European Society for Medical Oncology* 2016, **27**(7):1291-1298.
5. Garon EB, Rizvi NA, Hui R, Leighl N, Balmanoukian AS, Eder JP, Patnaik A, Aggarwal C, Gubens M, Horn L *et al*: **Pembrolizumab for the treatment of non-small-cell lung cancer**. *The New England journal of medicine* 2015, **372**(21):2018-2028.
6. Goldberg SB, Gettinger SN, Mahajan A, Chiang AC, Herbst RS, Sznol M, Tsiouris AJ, Cohen J, Vortmeyer A, Jilaveanu L *et al*: **Pembrolizumab for patients with melanoma or non-small-cell lung cancer and untreated brain metastases: early analysis of a non-randomised, open-label, phase 2 trial**. *The Lancet Oncology* 2016, **17**(7):976-983.
7. Herbst RS, Baas P, Kim DW, Felip E, Perez-Gracia JL, Han JY, Molina J, Kim JH, Arvis CD, Ahn MJ *et al*: **Pembrolizumab versus docetaxel for previously treated, PD-L1-positive, advanced non-small-cell lung cancer (KEYNOTE-010): a randomised controlled trial**. *Lancet (London, England)* 2016, **387**(10027):1540-1550.
8. Hui R, Garon EB, Goldman JW, Leighl NB, Hellmann MD, Patnaik A, Gandhi L, Eder JP, Ahn MJ, Horn L *et al*: **Pembrolizumab as first-line therapy for patients with PD-L1-positive advanced non-small cell lung cancer: a phase 1 trial**. *Annals of oncology : official journal of the European Society for Medical Oncology* 2017, **28**(4):874-881.
9. Langer CJ, Gadgeel SM, Borghaei H, Papadimitrakopoulou VA, Patnaik A, Powell SF, Gentzler RD, Martins RG, Stevenson JP, Jalal SI *et al*: **Carboplatin and pemetrexed with or without pembrolizumab for advanced, non-squamous non-small-cell lung cancer: a randomised, phase 2 cohort of the open-label KEYNOTE-021 study**. *The Lancet Oncology* 2016, **17**(11):1497-1508.
10. Reck M, Rodriguez-Abreu D, Robinson AG, Hui R, Csoszi T, Fulop A, Gottfried M, Peled N, Tafreshi A, Cuffe S *et al*: **Pembrolizumab versus Chemotherapy for PD-L1-Positive Non-Small-Cell Lung Cancer**. *The New England journal of medicine* 2016, **375**(19):1823-1833.
11. Roach C, Zhang N, Corigliano E, Jansson M, Toland G, Ponto G, Dolled-Filhart M, Emancipator K, Stanforth D, Kulangara K: **Development of a Companion Diagnostic PD-L1 Immunohistochemistry Assay for Pembrolizumab Therapy in Non-Small-cell Lung Cancer**. *Applied immunohistochemistry & molecular morphology : AIMM* 2016, **24**(6):392-397.
12. Shaverdian N, Lisberg AE, Bornazyan K, Veruttipong D, Goldman JW, Formenti SC, Garon EB, Lee P: **Previous radiotherapy and the clinical activity and toxicity of pembrolizumab in the treatment of non-small-cell lung cancer: a secondary analysis of the KEYNOTE-001 phase 1 trial**. *The Lancet Oncology* 2017, **18**(7):895-903.
13. Sul J, Blumenthal GM, Jiang X, He K, Keegan P, Pazdur R: **FDA Approval Summary: Pembrolizumab for the Treatment of Patients With Metastatic Non-Small Cell Lung Cancer Whose Tumors Express Programmed Death-Ligand 1**. *The oncologist* 2016, **21**(5):643-650.

# TOPIC: Non-Small Cell Lung Cancer + Durvalumab

1. Antonia S, Goldberg SB, Balmanoukian A, Chaft JE, Sanborn RE, Gupta A, Narwal R, Steele K, Gu Y, Karakunnel JJ *et al*: **Safety and antitumour activity of durvalumab plus tremelimumab in non-small cell lung cancer: a multicentre, phase 1b study**. *The Lancet Oncology* 2016, **17**(3):299-308.
2. Antonia SJ, Villegas A, Daniel D, Vicente D, Murakami S, Hui R, Yokoi T, Chiappori A, Lee KH, de Wit M *et al*: **Durvalumab after Chemoradiotherapy in Stage III Non-Small-Cell Lung Cancer**. *The New England journal of medicine* 2017, **377**(20):1919-1929.
3. Planchard D, Yokoi T, McCleod MJ, Fischer JR, Kim YC, Ballas M, Shi K, Soria JC: **A Phase III Study of Durvalumab (MEDI4736) With or Without Tremelimumab for Previously Treated Patients With Advanced NSCLC: Rationale and Protocol Design of the ARCTIC Study**. *Clinical lung cancer* 2016, **17**(3):232-236.e231.
4. Rebelatto MC, Midha A, Mistry A, Sabalos C, Schechter N, Li X, Jin X, Steele KE, Robbins PB, Blake-Haskins JA *et al*: **Development of a programmed cell death ligand-1 immunohistochemical assay validated for analysis of non-small cell lung cancer and head and neck squamous cell carcinoma**. *Diagnostic pathology* 2016, **11**(1):95.

# TOPIC: Non-Small Cell Lung Cancer + Atezolizumab

1. Peters S, Gettinger S, Johnson ML, Janne PA, Garassino MC, Christoph D, Toh CK, Rizvi NA, Chaft JE, Carcereny Costa E *et al*: **Phase II Trial of Atezolizumab As First-Line or Subsequent Therapy for Patients With Programmed Death-Ligand 1-Selected Advanced Non-Small-Cell Lung Cancer (BIRCH)**. *Journal of clinical oncology : official journal of the American Society of Clinical Oncology* 2017, **35**(24):2781-2789.
2. Rittmeyer A, Barlesi F, Waterkamp D, Park K, Ciardiello F, von Pawel J, Gadgeel SM, Hida T, Kowalski DM, Dols MC *et al*: **Atezolizumab versus docetaxel in patients with previously treated non-small-cell lung cancer (OAK): a phase 3, open-label, multicentre randomised controlled trial**. *Lancet (London, England)* 2017, **389**(10066):255-265.

# TOPIC: Non-Small Cell Lung Cancer + Vaccine

1. Alfonso S, Valdes-Zayas A, Santiesteban ER, Flores YI, Areces F, Hernandez M, Viada CE, Mendoza IC, Guerra PP, Garcia E *et al*: **A randomized, multicenter, placebo-controlled clinical trial of racotumomab-alum vaccine as switch maintenance therapy in advanced non-small cell lung cancer patients**. *Clinical cancer research : an official journal of the American Association for Cancer Research* 2014, **20**(14):3660-3671.
2. Atanackovic D, Altorki NK, Cao Y, Ritter E, Ferrara CA, Ritter G, Hoffman EW, Bokemeyer C, Old LJ, Gnjatic S: **Booster vaccination of cancer patients with MAGE-A3 protein reveals long-term immunological memory or tolerance depending on priming**. *Proceedings of the National Academy of Sciences of the United States of America* 2008, **105**(5):1650-1655
3. Barve M, Bender J, Senzer N, Cunningham C, Greco FA, McCune D, Steis R, Khong H, Richards D, Stephenson J *et al*: **Induction of immune responses and clinical efficacy in a phase II trial of IDM-2101, a 10-epitope cytotoxic T-lymphocyte vaccine, in metastatic non-small-cell lung cancer**. *Journal of clinical oncology : official journal of the American Society of Clinical Oncology* 2008, **26**(27):4418-4425.
4. Belani CP, Chakraborty BC, Modi RI, Khamar BM: **A randomized trial of TLR-2 agonist CADI-05 targeting desmocollin-3 for advanced non-small-cell lung cancer**. *Annals of oncology : official journal of the European Society for Medical Oncology* 2017, **28**(2):298-304.
5. Brunsvig PF, Kyte JA, Kersten C, Sundstrom S, Moller M, Nyakas M, Hansen GL, Gaudernack G, Aamdal S: **Telomerase peptide vaccination in NSCLC: a phase II trial in stage III patients vaccinated after chemoradiotherapy and an 8-year update on a phase I/II trial**. *Clinical cancer research : an official journal of the American Association for Cancer Research* 2011, **17**(21):6847-6857.
6. Butts C, Maksymiuk A, Goss G, Soulieres D, Marshall E, Cormier Y, Ellis PM, Price A, Sawhney R, Beier F *et al*: **Updated survival analysis in patients with stage IIIB or IV non-small-cell lung cancer receiving BLP25 liposome vaccine (L-BLP25): phase IIB randomized, multicenter, open-label trial**. *Journal of cancer research and clinical oncology* 2011, **137**(9):1337-1342.
7. Butts C, Murray RN, Smith CJ, Ellis PM, Jasas K, Maksymiuk A, Goss G, Ely G, Beier F, Soulieres D: **A multicenter open-label study to assess the safety of a new formulation of BLP25 liposome vaccine in patients with unresectable stage III non-small-cell lung cancer**. *Clinical lung cancer* 2010, **11**(6):391-395.
8. Butts C, Socinski MA, Mitchell PL, Thatcher N, Havel L, Krzakowski M, Nawrocki S, Ciuleanu TE, Bosquee L, Trigo JM *et al*: **Tecemotide (L-BLP25) versus placebo after chemoradiotherapy for stage III non-small-cell lung cancer (START): a randomised, double-blind, phase 3 trial**. *The Lancet Oncology* 2014, **15**(1):59-68.
9. Chaft JE, Litvak A, Arcila ME, Patel P, D'Angelo SP, Krug LM, Rusch V, Mattson A, Coeshott C, Park B *et al*: **Phase II study of the GI-4000 KRAS vaccine after curative therapy in patients with stage I-III lung adenocarcinoma harboring a KRAS G12C, G12D, or G12V mutation**. *Clinical lung cancer* 2014, **15**(6):405-410.
10. Dammeijer F, Lievense LA, Veerman GD, Hoogsteden HC, Hegmans JP, Arends LR, Aerts JG: **Efficacy of Tumor Vaccines and Cellular Immunotherapies in Non-Small-Cell Lung Cancer: A Systematic Review and Meta-Analysis**. *Journal of clinical oncology : official journal of the American Society of Clinical Oncology* 2016, **34**(26):3204-3212.
11. Ding M, Yang J: **Therapeutic vaccination for non-small-cell lung cancer: a meta-analysis**. *Medical oncology (Northwood, London, England)* 2014, **31**(4):928.
12. Garcia B, Neninger E, de la Torre A, Leonard I, Martinez R, Viada C, Gonzalez G, Mazorra Z, Lage A, Crombet T: **Effective inhibition of the epidermal growth factor/epidermal growth factor receptor binding by anti-epidermal growth factor antibodies is related to better survival in advanced non-small-cell lung cancer patients treated with the epidermal growth factor cancer vaccine**. *Clinical cancer research : an official journal of the American Association for Cancer Research* 2008, **14**(3):840-846.
13. Georgoulias V, Douillard JY, Khayat D, Manegold C, Rosell R, Rossi A, Menez-Jamet J, Iche M, Kosmatopoulos K, Gridelli C: **A multicenter randomized phase IIb efficacy study of Vx-001, a peptide-based cancer vaccine as maintenance treatment in advanced non-small-cell lung cancer: treatment rationale and protocol dynamics**. *Clinical lung cancer* 2013, **14**(4):461-465.
14. Giaccone G, Bazhenova LA, Nemunaitis J, Tan M, Juhasz E, Ramlau R, van den Heuvel MM, Lal R, Kloecker GH, Eaton KD *et al*: **A phase III study of belagenpumatucel-L, an allogeneic tumour cell vaccine, as maintenance therapy for non-small cell lung cancer**. *European journal of cancer (Oxford, England : 1990)* 2015, **51**(16):2321-2329.
15. Iversen TZ, Engell-Noerregaard L, Ellebaek E, Andersen R, Larsen SK, Bjoern J, Zeyher C, Gouttefangeas C, Thomsen BM, Holm B *et al*: **Long-lasting disease stabilization in the absence of toxicity in metastatic lung cancer patients vaccinated with an epitope derived from indoleamine 2,3 dioxygenase**. *Clinical cancer research : an official journal of the American Association for Cancer Research* 2014, **20**(1):221-232.
16. Katakami N, Hida T, Nokihara H, Imamura F, Sakai H, Atagi S, Nishio M, Kashii T, Satouchi M, Helwig C *et al*: **Phase I/II study of tecemotide as immunotherapy in Japanese patients with unresectable stage III non-small cell lung cancer**. *Lung cancer (Amsterdam, Netherlands)* 2017, **105**:23-30.
17. Kotsakis A, Papadimitraki E, Vetsika EK, Aggouraki D, Dermitzaki EK, Hatzidaki D, Kentepozidis N, Mavroudis D, Georgoulias V: **A phase II trial evaluating the clinical and immunologic response of HLA-A2(+) non-small cell lung cancer patients vaccinated with an hTERT cryptic peptide**. *Lung cancer (Amsterdam, Netherlands)* 2014, **86**(1):59-66.
18. Krug LM, Dao T, Brown AB, Maslak P, Travis W, Bekele S, Korontsvit T, Zakhaleva V, Wolchok J, Yuan J *et al*: **WT1 peptide vaccinations induce CD4 and CD8 T cell immune responses in patients with mesothelioma and non-small cell lung cancer**. *Cancer immunology, immunotherapy : CII* 2010, **59**(10):1467-1479.
19. Mitchell P, Thatcher N, Socinski MA, Wasilewska-Tesluk E, Horwood K, Szczesna A, Martin C, Ragulin Y, Zukin M, Helwig C *et al*: **Tecemotide in unresectable stage III non-small-cell lung cancer in the phase III START study: updated overall survival and biomarker analyses**. *Annals of oncology : official journal of the European Society for Medical Oncology* 2015, **26**(6):1134-1142.
20. Motohashi S, Nagato K, Kunii N, Yamamoto H, Yamasaki K, Okita K, Hanaoka H, Shimizu N, Suzuki M, Yoshino I *et al*: **A phase I-II study of alpha-galactosylceramide-pulsed IL-2/GM-CSF-cultured peripheral blood mononuclear cells in patients with advanced and recurrent non-small cell lung cancer**. *Journal of immunology (Baltimore, Md : 1950)* 2009, **182**(4):2492-2501.
21. Nemunaitis J, Nemunaitis M, Senzer N, Snitz P, Bedell C, Kumar P, Pappen B, Maples PB, Shawler D, Fakhrai H: **Phase II trial of Belagenpumatucel-L, a TGF-beta2 antisense gene modified allogeneic tumor vaccine in advanced non small cell lung cancer (NSCLC) patients**. *Cancer gene therapy* 2009, **16**(8):620-624.
22. Neninger E, Verdecia BG, Crombet T, Viada C, Pereda S, Leonard I, Mazorra Z, Fleites G, Gonzalez M, Wilkinson B *et al*: **Combining an EGF-based cancer vaccine with chemotherapy in advanced nonsmall cell lung cancer**. *Journal of immunotherapy (Hagerstown, Md : 1997)* 2009, **32**(1):92-99.
23. Neninger Vinageras E, de la Torre A, Osorio Rodriguez M, Catala Ferrer M, Bravo I, Mendoza del Pino M, Abreu Abreu D, Acosta Brooks S, Rives R, del Castillo Carrillo C *et al*: **Phase II randomized controlled trial of an epidermal growth factor vaccine in advanced non-small-cell lung cancer**. *Journal of clinical oncology : official journal of the American Society of Clinical Oncology* 2008, **26**(9):1452-1458.
24. Ohyanagi F, Horai T, Sekine I, Yamamoto N, Nakagawa K, Nishio M, Senger S, Morsli N, Tamura T: **Safety of BLP25 liposome vaccine (L-BLP25) in Japanese patients with unresectable stage III NSCLC after primary chemoradiotherapy: preliminary results from a Phase I/II study**. *Japanese journal of clinical oncology* 2011, **41**(5):718-722.
25. Perroud MW, Jr., Honma HN, Barbeiro AS, Gilli SC, Almeida MT, Vassallo J, Saad ST, Zambon L: **Mature autologous dendritic cell vaccines in advanced non-small cell lung cancer: a phase I pilot study**. *Journal of experimental & clinical cancer research : CR* 2011, **30**:65.
26. Quoix E, Lena H, Losonczy G, Forget F, Chouaid C, Papai Z, Gervais R, Ottensmeier C, Szczesna A, Kazarnowicz A *et al*: **TG4010 immunotherapy and first-line chemotherapy for advanced non-small-cell lung cancer (TIME): results from the phase 2b part of a randomised, double-blind, placebo-controlled, phase 2b/3 trial**. *The Lancet Oncology* 2016, **17**(2):212-223.
27. Quoix E, Ramlau R, Westeel V, Papai Z, Madroszyk A, Riviere A, Koralewski P, Breton JL, Stoelben E, Braun D *et al*: **Therapeutic vaccination with TG4010 and first-line chemotherapy in advanced non-small-cell lung cancer: a controlled phase 2B trial**. *The Lancet Oncology* 2011, **12**(12):1125-1133.
28. Ramlau R, Quoix E, Rolski J, Pless M, Lena H, Levy E, Krzakowski M, Hess D, Tartour E, Chenard MP *et al*: **A phase II study of Tg4010 (Mva-Muc1-Il2) in association with chemotherapy in patients with stage III/IV Non-small cell lung cancer**. *Journal of thoracic oncology : official publication of the International Association for the Study of Lung Cancer* 2008, **3**(7):735-744.
29. Rodriguez PC, Popa X, Martinez O, Mendoza S, Santiesteban E, Crespo T, Amador RM, Fleytas R, Acosta SC, Otero Y *et al*: **A Phase III Clinical Trial of the Epidermal Growth Factor Vaccine CIMAvax-EGF as Switch Maintenance Therapy in Advanced Non-Small Cell Lung Cancer Patients**. *Clinical cancer research : an official journal of the American Association for Cancer Research* 2016, **22**(15):3782-3790.
30. Rotonda C, Anota A, Mercier M, Bastien B, Lacoste G, Limacher JM, Quoix E, Bonnetain F: **Impact of TG4010 Vaccine on Health-Related Quality of Life in Advanced Non-Small-Cell Lung Cancer: Results of a Phase IIB Clinical Trial**. *PloS one* 2015, **10**(7):e0132568.
31. Sebastian M, Papachristofilou A, Weiss C, Fruh M, Cathomas R, Hilbe W, Wehler T, Rippin G, Koch SD, Scheel B *et al*: **Phase Ib study evaluating a self-adjuvanted mRNA cancer vaccine (RNActive(R)) combined with local radiation as consolidation and maintenance treatment for patients with stage IV non-small cell lung cancer**. *BMC cancer* 2014, **14**:748.
32. Skachkova OV, Khranovska NM, Gorbach OI, Svergun NM, Sydor RI, Nikulina VV: **Immunological markers of anti-tumor dendritic cells vaccine efficiency in patients with non-small cell lung cancer**. *Experimental oncology* 2013, **35**(2):109-113.
33. Sovenko VM, Khranovskaia NN, Ganul AV, Ganul VL, Grinevich Iu A, Orel VE, Skachkova OV, Svergun NN, Bororov LV, Borisiuk BO *et al*: **[About the impact of the dendritic cell autovaccine on the results of treatment of non-small-cell lung cancer patients]**. *Likars'ka sprava* 2013(6):68-72.
34. Stanford JL, Stanford CA, O'Brien ME, Grange JM: **Successful immunotherapy with Mycobacterium vaccae in the treatment of adenocarcinoma of the lung**. *European journal of cancer (Oxford, England : 1990)* 2008, **44**(2):224-227.
35. Suzuki H, Fukuhara M, Yamaura T, Mutoh S, Okabe N, Yaginuma H, Hasegawa T, Yonechi A, Osugi J, Hoshino M *et al*: **Multiple therapeutic peptide vaccines consisting of combined novel cancer testis antigens and anti-angiogenic peptides for patients with non-small cell lung cancer**. *Journal of translational medicine* 2013, **11**:97.
36. Takayama K, Sugawara S, Saijo Y, Maemondo M, Sato A, Takamori S, Harada T, Sasada T, Kakuma T, Kishimoto J *et al*: **Randomized Phase II Study of Docetaxel plus Personalized Peptide Vaccination versus Docetaxel plus Placebo for Patients with Previously Treated Advanced Wild Type EGFR Non-Small-Cell Lung Cancer**. *Journal of immunology research* 2016, **2016**:1745108.
37. Thatcher N, Heighway J: **Maintenance and consolidation therapy in patients with unresectable stage III/IV non-small cell lung cancer**. *The oncologist* 2010, **15**(10):1034-1042.
38. Ulloa-Montoya F, Louahed J, Dizier B, Gruselle O, Spiessens B, Lehmann FF, Suciu S, Kruit WH, Eggermont AM, Vansteenkiste J *et al*: **Predictive gene signature in MAGE-A3 antigen-specific cancer immunotherapy**. *Journal of clinical oncology : official journal of the American Society of Clinical Oncology* 2013, **31**(19):2388-2395.
39. Um SJ, Choi YJ, Shin HJ, Son CH, Park YS, Roh MS, Kim YS, Kim YD, Lee SK, Jung MH *et al*: **Phase I study of autologous dendritic cell tumor vaccine in patients with non-small cell lung cancer**. *Lung cancer (Amsterdam, Netherlands)* 2010, **70**(2):188-194.
40. Vansteenkiste J, Zielinski M, Linder A, Dahabreh J, Gonzalez EE, Malinowski W, Lopez-Brea M, Vanakesa T, Jassem J, Kalofonos H *et al*: **Adjuvant MAGE-A3 immunotherapy in resected non-small-cell lung cancer: phase II randomized study results**. *Journal of clinical oncology : official journal of the American Society of Clinical Oncology* 2013, **31**(19):2396-2403.
41. Wang M, Cao JX, Liu YS, Xu BL, Li D, Zhang XY, Li JL, Liu JL, Wang HB, Wang ZX: **Evaluation of tumour vaccine immunotherapy for the treatment of advanced non-small cell lung cancer: a systematic meta-analysis**. *BMJ open* 2015, **5**(4):e006321.
42. Wu YL, Park K, Soo RA, Sun Y, Tyroller K, Wages D, Ely G, Yang JC, Mok T: **INSPIRE: A phase III study of the BLP25 liposome vaccine (L-BLP25) in Asian patients with unresectable stage III non-small cell lung cancer**. *BMC cancer* 2011, **11**:430.
43. Yamada T, Terazaki Y, Sakamoto S, Yoshiyama K, Matsueda S, Komatsu N, Waki K, Yamada A, Kawahara A, Kage M *et al*: **Feasibility study of personalized peptide vaccination for advanced non-small cell lung cancer patients who failed two or more treatment regimens**. *International journal of oncology* 2015, **46**(1):55-62.
44. Yoshiyama K, Terazaki Y, Matsueda S, Shichijo S, Noguchi M, Yamada A, Mine T, Ioji T, Itoh K, Shirouzu K *et al*: **Personalized peptide vaccination in patients with refractory non-small cell lung cancer**. *International journal of oncology* 2012, **40**(5):1492-1500.
45. Zhong R, Teng J, Han B, Zhong H: **Dendritic cells combining with cytokine-induced killer cells synergize chemotherapy in patients with late-stage non-small cell lung cancer**. *Cancer immunology, immunotherapy : CII* 2011, **60**(10):1497-1502.
46. Zhu J, Li R, Tiselius E, Roudi R, Teghararian O, Suo C, Song H: **Immunotherapy (excluding checkpoint inhibitors) for stage I to III non-small cell lung cancer treated with surgery or radiotherapy with curative intent**. *The Cochrane database of systematic reviews* 2017, **12**:Cd011300.

# TOPIC: Non-Small Cell Lung Cancer + Immunotherapy

1. Blumenthal GM, Zhang L, Zhang H, Kazandjian D, Khozin S, Tang S, Goldberg K, Sridhara R, Keegan P, Pazdur R: **Milestone Analyses of Immune Checkpoint Inhibitors, Targeted Therapy, and Conventional Therapy in Metastatic Non-Small Cell Lung Cancer Trials: A Meta-analysis**. *JAMA oncology* 2017, **3**(8):e171029.
2. Chen R, Deng X, Wu H, Peng P, Wen B, Li F, Li F: **Combined immunotherapy with dendritic cells and cytokine-induced killer cells for malignant tumors: a systematic review and meta-analysis**. *International immunopharmacology* 2014, **22**(2):451-464.
3. Custodio A, de Castro J: **Strategies for maintenance therapy in advanced non-small cell lung cancer: current status, unanswered questions and future directions**. *Critical reviews in oncology/hematology* 2012, **82**(3):338-360.
4. Ebina T, Fujimiya Y: **Cell transfer regimens in patients with highly advanced surgically unresectable non-small cell lung cancer: significantly improved overall survival in patients with lower levels of serum immunosuppressive acidic protein**. *Lung cancer (Amsterdam, Netherlands)* 2008, **60**(2):246-251.
5. Ettinger DS, Wood DE, Aisner DL, Akerley W, Bauman J, Chirieac LR, D'Amico TA, DeCamp MM, Dilling TJ, Dobelbower M *et al*: **Non-Small Cell Lung Cancer, Version 5.2017, NCCN Clinical Practice Guidelines in Oncology**. *Journal of the National Comprehensive Cancer Network : JNCCN* 2017, **15**(4):504-535.
6. Franco-Molina MA, Mendoza-Gamboa E, Zapata-Benavides P, Vera-Garcia ME, Castillo-Tello P, Garcia de la Fuente A, Mendoza RD, Garza RG, Tamez-Guerra RS, Rodriguez-Padilla C: **IMMUNEPOTENT CRP (bovine dialyzable leukocyte extract) adjuvant immunotherapy: a phase I study in non-small cell lung cancer patients**. *Cytotherapy* 2008, **10**(5):490-496.
7. Gandini S, Massi D, Mandala M: **PD-L1 expression in cancer patients receiving anti PD-1/PD-L1 antibodies: A systematic review and meta-analysis**. *Critical reviews in oncology/hematology* 2016, **100**:88-98.
8. Gerber DE, Spigel DR, Giorgadze D, Shtivelband M, Ponomarova OV, Shan JS, Menander KB, Belani CP: **Docetaxel Combined With Bavituximab in Previously Treated, Advanced Nonsquamous Non-Small-Cell Lung Cancer**. *Clinical lung cancer* 2016, **17**(3):169-176.
9. Han RX, Liu X, Pan P, Jia YJ, Yu JC: **Effectiveness and safety of chemotherapy combined with dendritic cells co-cultured with cytokine-induced killer cells in the treatment of advanced non-small-cell lung cancer: a systematic review and meta-analysis**. *PloS one* 2014, **9**(9):e108958.
10. Hu RH, Shi SB, Qi JL, Tian J, Tang XY, Liu GF, Chang CX: **Pemetrexed plus dendritic cells as second-line treatment for patients with stage IIIB/IV non-small cell lung cancer who had treatment with TKI**. *Medical oncology (Northwood, London, England)* 2014, **31**(8):63.
11. Iliopoulou EG, Kountourakis P, Karamouzis MV, Doufexis D, Ardavanis A, Baxevanis CN, Rigatos G, Papamichail M, Perez SA: **A phase I trial of adoptive transfer of allogeneic natural killer cells in patients with advanced non-small cell lung cancer**. *Cancer immunology, immunotherapy : CII* 2010, **59**(12):1781-1789.
12. Iwai K, Soejima K, Kudoh S, Umezato Y, Kaneko T, Yoshimori K, Tokuda H, Yamaguchi T, Mizoo A, Setoguchi Y *et al*: **Extended survival observed in adoptive activated T lymphocyte immunotherapy for advanced lung cancer: results of a multicenter historical cohort study**. *Cancer immunology, immunotherapy : CII* 2012, **61**(10):1781-1790.
13. Kimura H, Iizasa T, Ishikawa A, Shingyouji M, Yoshino M, Kimura M, Inada Y, Matsubayashi K: **Prospective phase II study of post-surgical adjuvant chemo-immunotherapy using autologous dendritic cells and activated killer cells from tissue culture of tumor-draining lymph nodes in primary lung cancer patients**. *Anticancer research* 2008, **28**(2b):1229-1238.
14. Kimura H, Matsui Y, Ishikawa A, Nakajima T, Yoshino M, Sakairi Y: **Randomized controlled phase III trial of adjuvant chemo-immunotherapy with activated killer T cells and dendritic cells in patients with resected primary lung cancer**. *Cancer immunology, immunotherapy : CII* 2015, **64**(1):51-59.
15. Kurose K, Ohue Y, Wada H, Iida S, Ishida T, Kojima T, Doi T, Suzuki S, Isobe M, Funakoshi T *et al*: **Phase Ia Study of FoxP3+ CD4 Treg Depletion by Infusion of a Humanized Anti-CCR4 Antibody, KW-0761, in Cancer Patients**. *Clinical cancer research : an official journal of the American Association for Cancer Research* 2015, **21**(19):4327-4336.
16. Lasalvia-Prisco E, Goldschmidt P, Galmarini F, Cucchi S, Vazquez J, Aghazarian M, Lasalvia-Galante E, Golomar W, Gordon W: **Addition of an induction regimen of antiangiogenesis and antitumor immunity to standard chemotherapy improves survival in advanced malignancies**. *Medical oncology (Northwood, London, England)* 2012, **29**(5):3626-3633.
17. Lee CK, Man J, Lord S, Links M, Gebski V, Mok T, Yang JC: **Checkpoint Inhibitors in Metastatic EGFR-Mutated Non-Small Cell Lung Cancer-A Meta-Analysis**. *Journal of thoracic oncology : official publication of the International Association for the Study of Lung Cancer* 2017, **12**(2):403-407.
18. Li H, Wang C, Yu J, Cao S, Wei F, Zhang W, Han Y, Ren XB: **Dendritic cell-activated cytokine-induced killer cells enhance the anti-tumor effect of chemotherapy on non-small cell lung cancer in patients after surgery**. *Cytotherapy* 2009, **11**(8):1076-1083.
19. Li R, Wang C, Liu L, Du C, Cao S, Yu J, Wang SE, Hao X, Ren X, Li H: **Autologous cytokine-induced killer cell immunotherapy in lung cancer: a phase II clinical study**. *Cancer immunology, immunotherapy : CII* 2012, **61**(11):2125-2133.
20. Mi D, Ren W, Yang K: **Adoptive immunotherapy with interleukin-2 & induced killer cells in non-small cell lung cancer: A systematic review & meta-analysis**. *The Indian journal of medical research* 2016, **143**(Supplement):S1-s10.
21. Nakajima J, Murakawa T, Fukami T, Goto S, Kaneko T, Yoshida Y, Takamoto S, Kakimi K: **A phase I study of adoptive immunotherapy for recurrent non-small-cell lung cancer patients with autologous gammadelta T cells**. *European journal of cardio-thoracic surgery : official journal of the European Association for Cardio-thoracic Surgery* 2010, **37**(5):1191-1197.
22. Parkhurst MR, Yang JC, Langan RC, Dudley ME, Nathan DA, Feldman SA, Davis JL, Morgan RA, Merino MJ, Sherry RM *et al*: **T cells targeting carcinoembryonic antigen can mediate regression of metastatic colorectal cancer but induce severe transient colitis**. *Mol Ther* 2011, **19**(3):620-626.
23. Pujol JL, De Pas T, Rittmeyer A, Vallieres E, Kubisa B, Levchenko E, Wiesemann S, Masters GA, Shen R, Tjulandin SA *et al*: **Safety and Immunogenicity of the PRAME Cancer Immunotherapeutic in Patients with Resected Non-Small Cell Lung Cancer: A Phase I Dose Escalation Study**. *Journal of thoracic oncology : official publication of the International Association for the Study of Lung Cancer* 2016, **11**(12):2208-2217.
24. Pujol JL, Vansteenkiste JF, De Pas TM, Atanackovic D, Reck M, Thomeer M, Douillard JY, Fasola G, Potter V, Taylor P *et al*: **Safety and Immunogenicity of MAGE-A3 Cancer Immunotherapeutic with or without Adjuvant Chemotherapy in Patients with Resected Stage IB to III MAGE-A3-Positive Non-Small-Cell Lung Cancer**. *Journal of thoracic oncology : official publication of the International Association for the Study of Lung Cancer* 2015, **10**(10):1458-1467.
25. Qian H, Wang H, Guan X, Yi Z, Ma F: **Adoptive immunotherapy combined chemoradiotherapy for non-small-cell lung cancer: a meta-analysis**. *Anti-cancer drugs* 2016, **27**(5):433-438.
26. Ramalingam S, Crawford J, Chang A, Manegold C, Perez-Soler R, Douillard JY, Thatcher N, Barlesi F, Owonikoko T, Wang Y *et al*: **Talactoferrin alfa versus placebo in patients with refractory advanced non-small-cell lung cancer (FORTIS-M trial)**. *Annals of oncology : official journal of the European Society for Medical Oncology* 2013, **24**(11):2875-2880.
27. Ratto GB, Costa R, Maineri P, Alloisio A, Piras MT, D'Agostino A, Tripodi G, Rivabella L, Dozin B, Bruzzi P *et al*: **Neo-adjuvant chemo/immunotherapy in the treatment of stage III (N2) non-small cell lung cancer: a phase I/II pilot study**. *International journal of immunopathology and pharmacology* 2011, **24**(4):1005-1016.
28. Ridolfi L, Bertetto O, Santo A, Naglieri E, Lopez M, Recchia F, Lissoni P, Galliano M, Testore F, Porta C *et al*: **Chemotherapy with or without low-dose interleukin-2 in advanced non-small cell lung cancer: results from a phase III randomized multicentric trial**. *International journal of oncology* 2011, **39**(4):1011-1017.
29. Sakamoto M, Nakajima J, Murakawa T, Fukami T, Yoshida Y, Murayama T, Takamoto S, Matsushita H, Kakimi K: **Adoptive immunotherapy for advanced non-small cell lung cancer using zoledronate-expanded gammadeltaTcells: a phase I clinical study**. *Journal of immunotherapy (Hagerstown, Md : 1997)* 2011, **34**(2):202-211.
30. Shi SB, Ma TH, Li CH, Tang XY: **Effect of maintenance therapy with dendritic cells: cytokine-induced killer cells in patients with advanced non-small cell lung cancer**. *Tumori* 2012, **98**(3):314-319.
31. Sun Z, Shi L, Zhang H, Shao Y, Wang Y, Lin Y, Li X, Bai C: **Immune modulation and safety profile of adoptive immunotherapy using expanded autologous activated lymphocytes against advanced cancer**. *Clinical immunology (Orlando, Fla)* 2011, **138**(1):23-32.
32. Thongprasert S, Yang PC, Lee JS, Soo R, Gruselle O, Myo A, Louahed J, Lehmann FF, Brichard VG, Coche T: **The prevalence of expression of MAGE-A3 and PRAME tumor antigens in East and South East Asian non-small cell lung cancer patients**. *Lung cancer (Amsterdam, Netherlands)* 2016, **101**:137-144.
33. van den Heuvel MM, Verheij M, Boshuizen R, Belderbos J, Dingemans AM, De Ruysscher D, Laurent J, Tighe R, Haanen J, Quaratino S: **NHS-IL2 combined with radiotherapy: preclinical rationale and phase Ib trial results in metastatic non-small cell lung cancer following first-line chemotherapy**. *Journal of translational medicine* 2015, **13**:32.
34. Vansteenkiste JF, Cho BC, Vanakesa T, De Pas T, Zielinski M, Kim MS, Jassem J, Yoshimura M, Dahabreh J, Nakayama H *et al*: **Efficacy of the MAGE-A3 cancer immunotherapeutic as adjuvant therapy in patients with resected MAGE-A3-positive non-small-cell lung cancer (MAGRIT): a randomised, double-blind, placebo-controlled, phase 3 trial**. *The Lancet Oncology* 2016, **17**(6):822-835.
35. Wang J, Zou ZH, Xia HL, He JX, Zhong NS, Tao AL: **Strengths and weaknesses of immunotherapy for advanced non-small-cell lung cancer: a meta-analysis of 12 randomized controlled trials**. *PloS one* 2012, **7**(3):e32695.
36. Wang M, Cao JX, Pan JH, Liu YS, Xu BL, Li D, Zhang XY, Li JL, Liu JL, Wang HB *et al*: **Adoptive immunotherapy of cytokine-induced killer cell therapy in the treatment of non-small cell lung cancer**. *PloS one* 2014, **9**(11):e112662.
37. Wang S, Wang Z: **Efficacy and safety of dendritic cells co-cultured with cytokine-induced killer cells immunotherapy for non-small-cell lung cancer**. *International immunopharmacology* 2015, **28**(1):22-28.
38. Wu C, Jiang J, Shi L, Xu N: **Prospective study of chemotherapy in combination with cytokine-induced killer cells in patients suffering from advanced non-small cell lung cancer**. *Anticancer research* 2008, **28**(6b):3997-4002.
39. Yang L, Ren B, Li H, Yu J, Cao S, Hao X, Ren X: **Enhanced antitumor effects of DC-activated CIKs to chemotherapy treatment in a single cohort of advanced non-small-cell lung cancer patients**. *Cancer immunology, immunotherapy : CII* 2013, **62**(1):65-73.
40. Yang YJ, Park JC, Kim HK, Kang JH, Park SY: **A trial of autologous ex vivo-expanded NK cell-enriched lymphocytes with docetaxel in patients with advanced non-small cell lung cancer as second- or third-line treatment: phase IIa study**. *Anticancer research* 2013, **33**(5):2115-2122.
41. Zeng Y, Ruan W, He J, Zhang J, Liang W, Chen Y, He Q, He J: **Adoptive Immunotherapy in Postoperative Non-Small-Cell Lung Cancer: A Systematic Review and Meta-Analysis**. *PloS one* 2016, **11**(9):e0162630.
42. Zhao P, Bu X, Wei X, Sun W, Xie X, Li C, Guo Q, Zhu D, Wei X, Gao D: **Dendritic cell immunotherapy combined with cytokine-induced killer cells promotes skewing toward Th2 cytokine profile in patients with metastatic non-small cell lung cancer**. *International immunopharmacology* 2015, **25**(2):450-456.
43. Zhao Z, Su Z, Zhang W, Luo M, Wang H, Huang L: **A randomized study comparing the effectiveness of microwave ablation radioimmunotherapy and postoperative adjuvant chemoradiation in the treatment of non-small cell lung cancer**. *Journal of BUON : official journal of the Balkan Union of Oncology* 2016, **21**(2):326-332.
44. Zhong R, Han B, Zhong H: **A prospective study of the efficacy of a combination of autologous dendritic cells, cytokine-induced killer cells, and chemotherapy in advanced non-small cell lung cancer patients**. *Tumour biology : the journal of the International Society for Oncodevelopmental Biology and Medicine* 2014, **35**(2):987-994.

# TOPIC: Non-Small Cell Lung Cancer + PD-1/PD-L1

1. Abdel-Rahman O: **Correlation between PD-L1 expression and outcome of NSCLC patients treated with anti-PD-1/PD-L1 agents: A meta-analysis**. *Critical reviews in oncology/hematology* 2016, **101**:75-85.
2. Aguiar PN, Jr., Santoro IL, Tadokoro H, de Lima Lopes G, Filardi BA, Oliveira P, Mountzios G, de Mello RA: **The role of PD-L1 expression as a predictive biomarker in advanced non-small-cell lung cancer: a network meta-analysis**. *Immunotherapy* 2016, **8**(4):479-488.
3. Fehrenbacher L, Spira A, Ballinger M, Kowanetz M, Vansteenkiste J, Mazieres J, Park K, Smith D, Artal-Cortes A, Lewanski C *et al*: **Atezolizumab versus docetaxel for patients with previously treated non-small-cell lung cancer (POPLAR): a multicentre, open-label, phase 2 randomised controlled trial**. *Lancet (London, England)* 2016, **387**(10030):1837-1846.
4. Gulley JL, Rajan A, Spigel DR, Iannotti N, Chandler J, Wong DJL, Leach J, Edenfield WJ, Wang D, Grote HJ *et al*: **Avelumab for patients with previously treated metastatic or recurrent non-small-cell lung cancer (JAVELIN Solid Tumor): dose-expansion cohort of a multicentre, open-label, phase 1b trial**. *The Lancet Oncology* 2017, **18**(5):599-610.
5. Herbst RS, Soria JC, Kowanetz M, Fine GD, Hamid O, Gordon MS, Sosman JA, McDermott DF, Powderly JD, Gettinger SN *et al*: **Predictive correlates of response to the anti-PD-L1 antibody MPDL3280A in cancer patients**. *Nature* 2014, **515**(7528):563-567.
6. Hirsch FR, McElhinny A, Stanforth D, Ranger-Moore J, Jansson M, Kulangara K, Richardson W, Towne P, Hanks D, Vennapusa B *et al*: **PD-L1 Immunohistochemistry Assays for Lung Cancer: Results from Phase 1 of the Blueprint PD-L1 IHC Assay Comparison Project**. *Journal of thoracic oncology : official publication of the International Association for the Study of Lung Cancer* 2017, **12**(2):208-222.
7. Hu XY, Zhang W, Hu Y, Zhang Y, Gong R, Liang JY, Liu L: **A meta-analysis reveals prognostic role of programmed death ligand-1 in Asian patients with non-small cell lung cancer**. *Journal of Huazhong University of Science and Technology Medical sciences = Hua zhong ke ji da xue xue bao Yi xue Ying De wen ban = Huazhong keji daxue xuebao Yixue Yingdewen ban* 2016, **36**(3):313-320.
8. Li D, Zhu X, Wang H, Li N: **Association between PD-L1 expression and driven gene status in NSCLC: A meta-analysis**. *European journal of surgical oncology : the journal of the European Society of Surgical Oncology and the British Association of Surgical Oncology* 2017, **43**(7):1372-1379.
9. Sun X, Roudi R, Chen S, Fan B, Li HJ, Zhou M, Li X, Li B: **Immune-related adverse events associated with PD-1 and PD-L1 inhibitors for nonsmall cell lung cancer: Protocol for a systematic review and meta-analysis**. *Medicine* 2017, **96**(44):e8407.
10. Wang A, Wang HY, Liu Y, Zhao MC, Zhang HJ, Lu ZY, Fang YC, Chen XF, Liu GT: **The prognostic value of PD-L1 expression for non-small cell lung cancer patients: a meta-analysis**. *European journal of surgical oncology : the journal of the European Society of Surgical Oncology and the British Association of Surgical Oncology* 2015, **41**(4):450-456.
11. Yang H, Chen H, Luo S, Li L, Zhou S, Shen R, Lin H, Xie X: **The correlation between programmed death-ligand 1 expression and driver gene mutations in NSCLC**. *Oncotarget* 2017, **8**(14):23517-23528.
12. Zhou GW, Xiong Y, Chen S, Xia F, Li Q, Hu J: **Anti-PD-1/PD-L1 antibody therapy for pretreated advanced nonsmall-cell lung cancer: A meta-analysis of randomized clinical trials**. *Medicine* 2016, **95**(35):e4611.
13. Zhu L, Jing S, Wang B, Wu K, Shenglin MA, Zhang S: **Anti-PD-1/PD-L1 Therapy as a Promising Option for Non-Small Cell Lung Cancer: a Single arm Meta-Analysis**. *Pathology oncology research : POR* 2016, **22**(2):331-339.

# TOPIC: Non-Small Cell Lung Cancer + Combination Immunotherapy

No original entries

# TOPIC: Non-Small Cell Lung Cancer + Immunotherapy Adverse Events

1. Kato T, Masuda N, Nakanishi Y, Takahashi M, Hida T, Sakai H, Atagi S, Fujita S, Tanaka H, Takeda K *et al*: **Nivolumab-induced interstitial lung disease analysis of two phase II studies patients with recurrent or advanced non-small-cell lung cancer**. *Lung cancer (Amsterdam, Netherlands)* 2017, **104**:111-118.
2. Khunger M, Rakshit S, Pasupuleti V, Hernandez AV, Mazzone P, Stevenson J, Pennell NA, Velcheti V: **Incidence of Pneumonitis With Use of Programmed Death 1 and Programmed Death-Ligand 1 Inhibitors in Non-Small Cell Lung Cancer: A Systematic Review and Meta-Analysis of Trials**. *Chest* 2017, **152**(2):271-281.
3. Nishino M, Giobbie-Hurder A, Hatabu H, Ramaiya NH, Hodi FS: **Incidence of Programmed Cell Death 1 Inhibitor-Related Pneumonitis in Patients With Advanced Cancer: A Systematic Review and Meta-analysis**. *JAMA oncology* 2016, **2**(12):1607-1616.
4. Osorio JC, Ni A, Chaft JE, Pollina R, Kasler MK, Stephens D, Rodriguez C, Cambridge L, Rizvi H, Wolchok JD *et al*: **Antibody-mediated thyroid dysfunction during T-cell checkpoint blockade in patients with non-small-cell lung cancer**. *Annals of oncology : official journal of the European Society for Medical Oncology* 2017, **28**(3):583-589.
